# Sensory and Cognitive Modulation of Saltiness Processing in Everyday Food: Evidence from Two fNIRS Experiments

**DOI:** 10.3390/foods15213916

**Published:** 2026-11-01

**Authors:** Yinan Jiang, Nobuyuki Sakai

**Affiliations:** Department of Psychology, Graduate School of Arts and Letters, Tohoku University, Sendai 980-8576, Japan; yinan.jiang.a2@tohoku.ac.jp

**Keywords:** perceived saltiness, saltiness-enhancing effects, sensory factors, cognitive factors, umami substances, odors, package cues, mismatch

## Abstract

Maintaining the palatability of low-salt foods while reducing salt intake is an important challenge in the promotion of such products. Reducing salt content may decrease food palatability and may also lead consumers to form negative taste expectations before actually tasting the food. Two experiments using functional near-infrared spectroscopy (fNIRS), each involving 30 participants, examined whether umami substances and olfactory cues could act as sensory factors, and package cues as a cognitive factor, to influence perceived saltiness in soups with complex flavor profiles. Experiment 1 showed no significant saltiness-enhancing effects for the tested saltiness enhancers. Descriptive rating patterns highlight the need to further examine the potential role of food flavor context. In Experiment 2, no significant effects of package cues on saltiness or palatability ratings were detected, while exploratory neural findings suggested condition-related response differences, including possible sensitivity to package–soup mismatch. Overall, these findings motivate further investigation of sensory and cognitive factors in the evaluation of everyday foods with complex flavor profiles.

## 1. Introduction

Japan is one of the countries that have already implemented voluntary measures to reduce sodium intake [1]. However, the 2024 National Health and Nutrition Survey showed that the average salt intake among Japanese adults aged ≥ 20 years was 9.6 g/day [2], which is above both the target of 7 g/day set by the Third Term Health Japan 21 initiative [3] and the World Health Organization recommendation of 5 g/day [4]. Therefore, promoting practical consumer-oriented salt reduction initiatives is essential.

However, promoting low-salt foods is often challenging because reducing salt content can affect various sensory experiences beyond saltiness, leading to a reduction in overall flavor perception [5,6]. For example, salt suppresses bitterness, enhances sweetness, improves water retention in meat products, and modifies bread texture [7,8,9,10]. Consequently, although consumers are aware of the health risks associated with excessive sodium intake, most of them still prefer high-sodium foods [5].

In addition to the inherent loss of flavor caused by salt reduction, consumer attitudes toward low-salt foods may also be influenced by cognitive assumptions stemming from label information and existing expectations. Previous research has shown that health labeling or low-salt claims provide consumers with a negative prior expectation of reduced taste quality, even before consumption, which shapes subsequent taste perception [11]. However, negative attitudes toward low-salt foods do not necessarily reflect an inherent lack of taste acceptability. Girgis et al. [12] showed that a gradual 25% reduction in the sodium content of bread over six weeks was generally undetected by consumers while maintaining consumer acceptance.

Therefore, the effective promotion of low-salt products requires both the improvement of product-level sensory properties to maintain palatability and a better understanding of consumers’ subjective cognition and feelings to facilitate the actual and long-term consumption of low-salt food. Numerous studies [13,14,15,16,17,18,19] have proposed various approaches for enhancing saltiness or improving palatability and have combined sensory assessments with neurophysiological measures to investigate the mechanisms underlying these effects. However, most of these studies [13,14,15,16] used simplified model solutions, such as sodium chloride (NaCl) solutions, rather than everyday foods. These methods may limit our understanding of consumer perceptions in the context of food consumption. Moreover, many studies [13,15,16,17,18,19] have employed neural measurement techniques such as electroencephalography (EEG) and functional magnetic resonance imaging (fMRI), which have provided important insights into the neural mechanisms of taste and flavor perception. However, these methods are inherently sensitive to motion artifacts, and experimental paradigms often impose considerable constraints on participants’ movement and stimulus presentation.

Functional near-infrared spectroscopy (fNIRS) may offer a complementary approach to examine taste-related neural processing in a more ecologically relevant setting, particularly when larger amounts of food need to be consumed, and the task involves a certain degree of movement. fNIRS is a noninvasive neuroimaging technique that uses near-infrared light to measure changes in hemoglobin concentration in the cerebral cortex. fNIRS is relatively tolerant to motion artifacts and has low sensitivity to body movement [20], making it suitable for measuring neural activity during food intake [14,21,22,23].

Therefore, two experiments were conducted to examine the effects of sensory and cognitive factors, respectively, on saltiness perception in commercially available foods. fNIRS was used to assess the associated neural response patterns during soup consumption in a setting with relatively high ecological relevance.

Umami substances and olfactory cues are widely used as salt-perception enhancers in food products. Onuma et al. [14] combined sensory evaluation with fNIRS to examine the effect of monosodium glutamate (MSG) addition or the presence of soy sauce odor on the perceived saltiness of NaCl solutions and associated neural responses. The time–intensity (TI) evaluation results showed that both MSG addition and soy sauce odor significantly increased the perceived saltiness. The fNIRS results further indicated that both MSG and soy sauce odor enhanced the hemodynamic responses in the core regions of the frontal operculum (Brodmann area (BA)44 and BA45, respectively). Following the procedure described by Onuma et al. [14], in Experiment 1, we chose saltiness enhancers whose effectiveness has been demonstrated in previous studies: soy sauce odor and umami substances [24,25], and bacon odor [15,26]. We examined the saltiness-enhancing effects of these substances in everyday foods and the neural responses associated with these effects.

To reduce the potential influence of sodium ions, we did not use MSG, the most common umami substance. Instead, we used monopotassium glutamate (MPG), which contains no sodium ions but has a slightly different taste from MSG. Given that inosine monophosphate (IMP) can produce a synergistic umami-enhancing effect when combined with glutamate [27,28], we combined MPG with a small amount of IMP to enhance the overall umami intensity of the sample.

Food package cues conveying different salt content information were employed as cognitive factors in Experiment 2 to examine whether the match between packaging information and actual taste input influences subjective food evaluations and associated neural responses.

### Hypotheses

**H1 (Experiment 1).**  *Olfactory cues and umami substances enhance the perceived saltiness of foods with complex flavors and are associated with different patterns of neural involvement.*

Umami substances influence taste perception through direct gustatory stimulation, whereas olfactory cues influence taste perception through cross-modal processing. Furthermore, an EEG study [17] found that odor-induced saltiness enhancement was associated with delayed P3 latency without significant changes in the early P1 component, suggesting the involvement of later, higher-order integrative processing. These findings suggest that olfactory cues may enhance perceived saltiness through top-down modulation.

**H2 (Experiment 1).**  *The effectiveness of saltiness enhance* *rs varies depending on the food flavor profile, particularly for olfactory cues.*

Previous studies have suggested that congruency between olfactory and gustatory information can influence cross-modal integration [29,30]. This indicates that the effect of an added olfactory cue may not be uniform across foods with different flavor profiles. Accordingly, its saltiness-enhancing effect may vary depending on the specific flavor profile of the food in which it is present.

**H3 (Experiment 2).**  *Packag* *e cues influence saltiness evaluations and neural responses in cortical regions associated with higher-order cognitive processing, with these responses potentially modulated by whether the packaging information matches the actual salt content of the food.*

Previous studies have shown that salt-related labels can alter perceived saltiness and subsequent salt-use behavior [11], while brand information can influence behavioral preferences and the associated neural responses [31]. These findings suggest that packaging information may influence evaluation at both behavioral and neural levels.

## 2. Materials and Methods

### 2.1. Experiment 1

#### 2.1.1. Participants

Thirty Japanese university students (15 males and 15 females; *M*_age_ = 21.83, *SD* = 1.26) participated in Experiment 1. An a priori power analysis was conducted using G*Power 3.1.9.7 for a within-subject repeated-measures design with five conditions. Assuming an effect size of *f* = 0.25, α = 0.05, and a power of 0.80, the required sample size was estimated to be 21 participants. Therefore, a total of 30 participants were recruited to ensure an adequate sample size. All participants reported being in good health with normal taste and olfactory functions and were instructed to refrain from eating or drinking for at least 2 h before the experiment. The experiment was conducted in a psychology laboratory. Before data collection, the experimental procedures and safety of the fNIRS measurements were explained, and practice trials were conducted to familiarize the participants with the task. The participants were informed that they could withdraw from the experiment at any time if they experienced discomfort during the session. Written informed consent was obtained from all participants. The study protocol was approved by the Ethics Committee of the Kawauchi Minami Campus, Tohoku University (approval no. [2021-001]).

#### 2.1.2. Stimuli and Experimental Design

Four commercially available instant soups that are widely known in Japan were used as base taste stimuli: regular egg soup (Knorr^®^ Fluffy Egg Soup, Ajinomoto Co., Inc., Tokyo, Japan), low-salt egg soup (Knorr^®^ Fluffy Egg Soup Reduced Salt, Ajinomoto Co., Inc., Tokyo, Japan), regular corn soup (Knorr^®^ Cup Soup Corn Cream, Ajinomoto Co., Inc., Tokyo, Japan), and low-salt corn soup (Knorr^®^ Cup Soup Corn Cream Reduced Salt, Ajinomoto Co., Inc., Tokyo, Japan). For the low-salt egg and corn soups, three additional soup types were prepared with the addition of saltiness enhancers: MPG + IMP, soy sauce odor, and bacon odor. Consequently, five soup types were prepared separately for the egg and corn soups: regular soup, low-salt soup, low-salt soup + MPG (L-glutamic acid monopotassium salt monohydrate, Nacalai Tesque, Kyoto, Japan) + IMP (inosine 5′-monophosphate disodium salt hydrate, Tokyo Chemical Industry, Tokyo, Japan), low-salt soup + soy sauce odor (food flavor, YAMAHO Co Ltd., Kyoto, Japan) and low-salt soup + bacon odor (food flavor, YAMAHO, Co., Ltd., Kyoto, Japan) (Table 1). The amounts of these additional sensory cues were determined through pretesting. For the odors, three candidate levels were established, with the bacon and soy sauce odors judged by subjects to have approximately comparable perceived intensities at each level. These judgments were made without a formal rating scale. For MPG and IMP, several candidate compositions were selected with reference to a previous study using mice as an animal model [32]. All candidate samples, including the MPG + IMP and odor-enhanced samples, were then evaluated in a preliminary study with human participants. The final compositions were selected based on their similarity in taste to the regular soup and the absence of noticeable unpleasantness. These five soup types served as the experimental conditions in a single-factor within-subject design for both egg and corn soups, allowing comparison of perceived saltiness and palatability across conditions.

**Figure 1:**
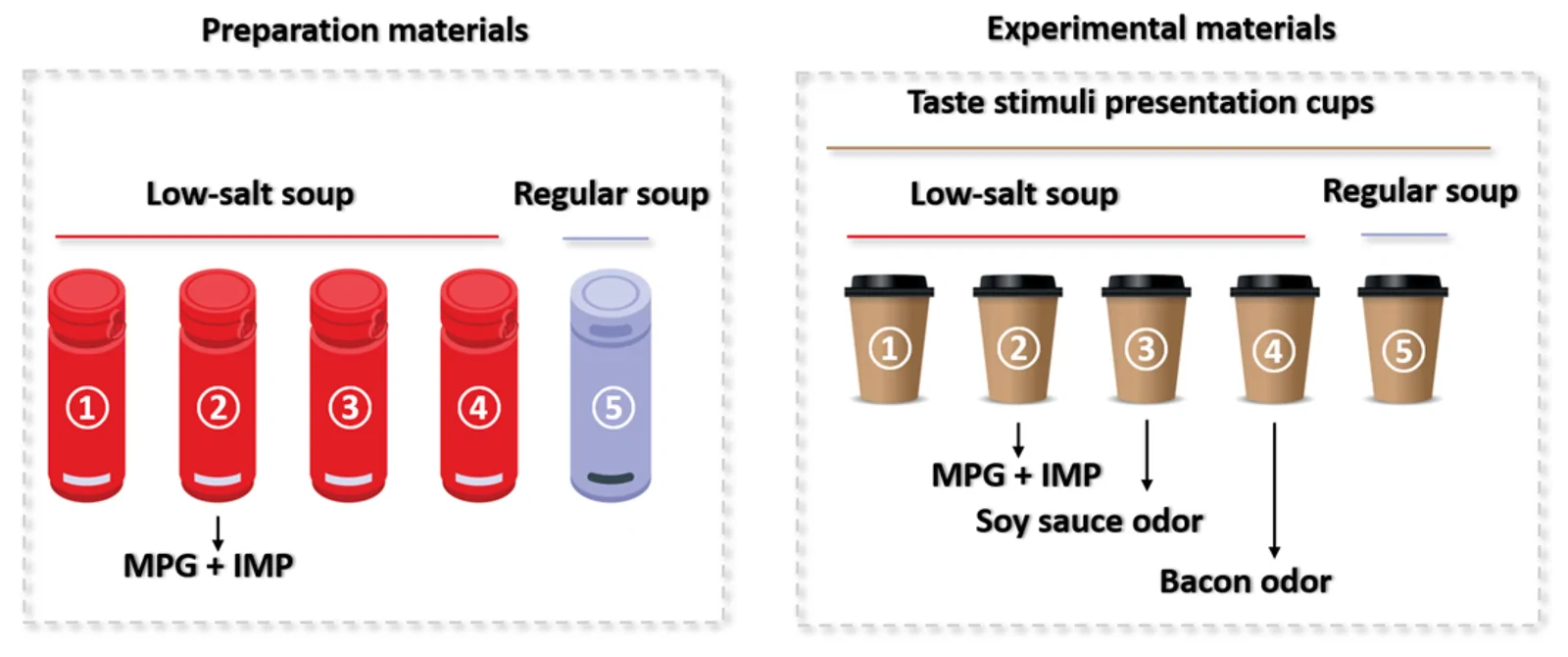
Schematic illustration of the preparation and presentation of the soups in Experiment 1. For each soup category (egg soup and corn soup), five soup types were prepared as experimental conditions: low-salt soup, low-salt soup with added MPG and IMP, low-salt soup with soy sauce odor, low-salt soup with bacon odor, and regular soup. The prepared samples were transferred to cups for presentation during the experiment.

To prepare the soup samples, one soup packet was used as a single unit and reconstituted with the manufacturer-recommended volume of water (see Table 1). All soups were prepared in vacuum-insulated containers, reconstituted with water at 90 °C, and stirred until completely dissolved (Figure 1).

For the low-salt soup samples containing umami saltiness enhancers, MPG was added at 0.42% (*w*/*v*), and IMP was added at 0.12% (*w*/*v*) to both the egg and corn soups, with the amounts adjusted according to the volume of water used. The temperature of the soup samples was measured using a digital probe thermometer. When the soup sample temperature dropped to 60 °C, the lid of the vacuum-insulated container was immediately tightened, and the container was placed in a constant-temperature incubator to maintain its temperature until the soup was presented to the participants.

The odor presentation method used in our previous study [14] was adopted in this study. The commercial bacon and soy sauce flavoring solutions were used as supplied, without dilution. Before the experiment, 20 μL of bacon flavoring solution or 5 μL of soy sauce flavoring solution was added dropwise onto 3 cm × 3 cm cotton pads using a pipette. The cotton pads were secured to the inner side of the paper cup lids. Three small holes were pre-drilled into the paper cup lids to allow the participants to better perceive the odors while consuming the soup samples. Paper cup lids with cotton pads treated with the different flavorings were stored separately to prevent cross-contamination and volatilization of the odors.

For each trial, 20 mL of the prepared soup sample was placed in a paper cup with a lid and presented to the participants. Low-salt soups with bacon or soy sauce odors were presented in cups fitted with lids preloaded with flavoring-treated cotton pads. All other conditions used identical lids with the same three pre-drilled holes but without cotton pads. The participants were instructed to consume the soup through openings in the paper cup’s lid.

#### 2.1.3. Procedure

**Figure 2:**
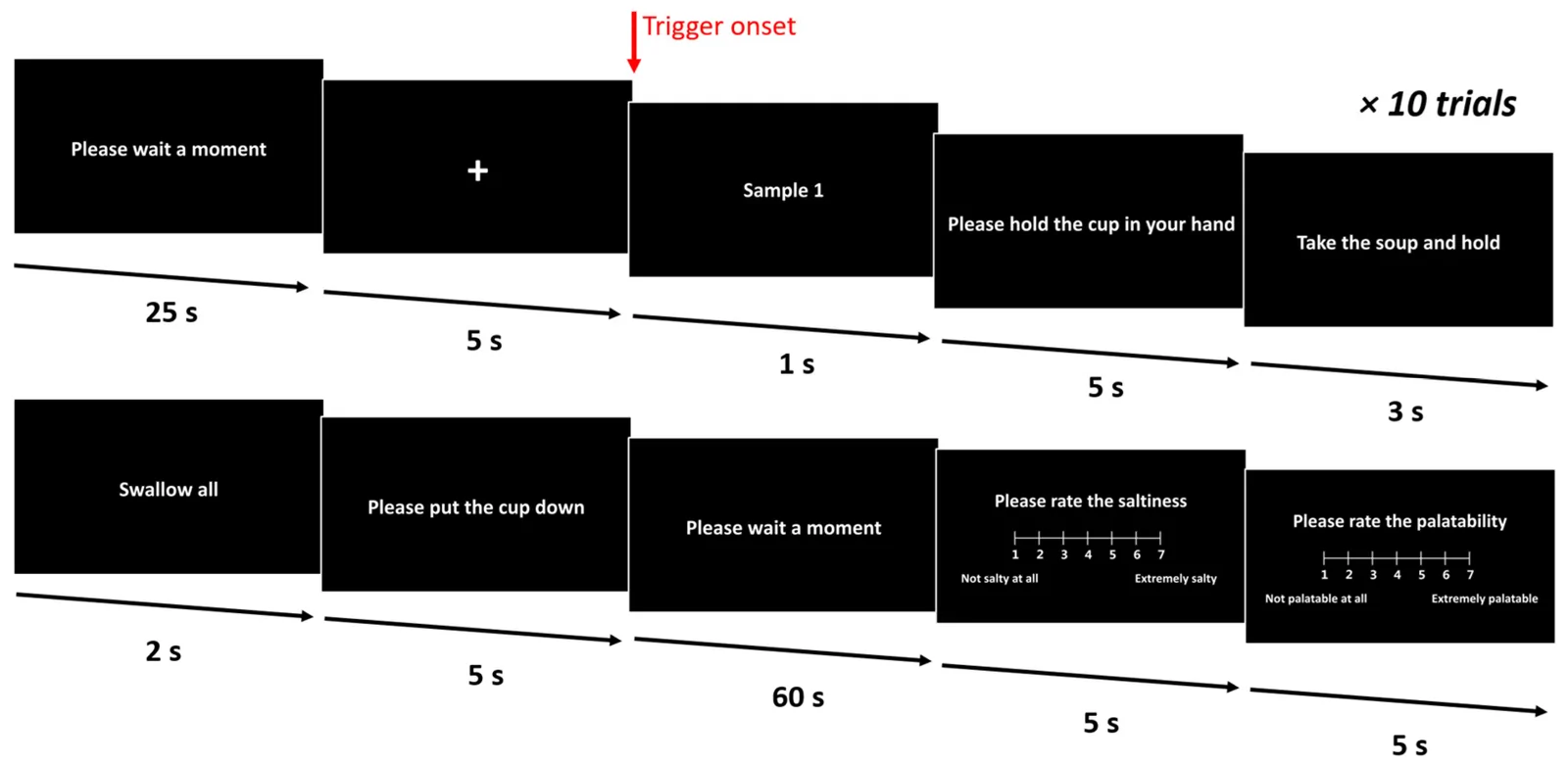
Flow chart of the procedure for Experiment 1. Each trial began with a 25 s rest period followed by a 5 s fixation period. After trigger onset, the sample number was presented, and participants followed the on-screen instructions to hold the cup, hold some soup in their mouths, swallow the soup, and then put the cup down. Following a 60 s post-consumption period, participants rated the perceived saltiness and palatability of the soup. Each participant completed a total of 10 trials.

Figure 2 illustrates the procedure used in Experiment 1. At the beginning of each trial, the participants were instructed to rest for 25 s, allowing the hemodynamic response to return to baseline before the stimulus was presented. The participants were then instructed to focus on a fixation cross (+) displayed on the screen for 5 s, followed by a sample number cue for 1 s. The onset of the sample-number cue was used as a trigger to extract condition-specific hemodynamic responses. The participants were then instructed to pick up and hold the cup in their hands for 5 s while preparing to drink. Subsequently, the participants were instructed to take some soup into their mouths and hold it for 3 s until the swallowing instructions appeared. The swallowing instructions were presented for 2 s. Afterward, the participants were instructed to place the cup down within 5 s. A 60 s period followed to allow for the recording of gustatory responses and for neural activity to return to baseline. After this period, the participants were asked to rate the saltiness and palatability (i.e., how good the soup tastes) of the soup using 7-point Likert scales ranging from 1 (not salty or palatable at all) to 7 (extremely salty or palatable). The interval between successive sample presentations was approximately 1.5 min, which helped reduce residual taste stimulation and sensory adaptation across trials to some extent.

The experiment consisted of ten conditions, with five conditions for each soup type. Egg and corn soups were presented in separate blocks. Within each block, low-salt soup was presented first, and regular soup was presented fifth. The three remaining conditions—low-salt soup with MPG + IMP, low-salt soup with bacon odor, and low-salt soup with soy sauce odor were presented in positions 2–4 using prespecified orders across participants. Half of the participants completed the egg soup block first, and the other half completed the corn soup block first (The presentation orders and participant allocation are shown in Supplementary Figure S1). Before the experiment, the participants were informed which soup type would be presented first; therefore, they knew the soup category to expect in each block. However, they were not informed of the order of experimental conditions within each block. Each condition was presented only once to capture the participants’ initial responses and to minimize potential learning and condition-specific anticipation effects arising from repeated exposure.

#### 2.1.4. fNIRS Data Acquisition and Channel Localization

Hemodynamic data were recorded using a LIGHTNIRS system (Shimadzu Corporation, Kyoto, Japan) [33] at a sampling rate of 13.3 Hz with three wavelengths of NIR light: 780, 805, and 830 nm. Short-separation channels were not available in the system used in the present study.

The optode set consisted of eight emitters and eight detectors, forming 20 channels. To standardize optode placement across participants, the fNIRS cap was positioned based on the international 10–20 system [34]. Four key anatomical landmarks, namely the nasion (Nz), inion (Iz), and two preauricular points (AR and AL), were determined. The vertex (Cz) was defined as the intersection of the midpoints of the Nz–Iz and AL–AR lines, and the top reference point of the cap was aligned with Cz.

**Figure 3:**
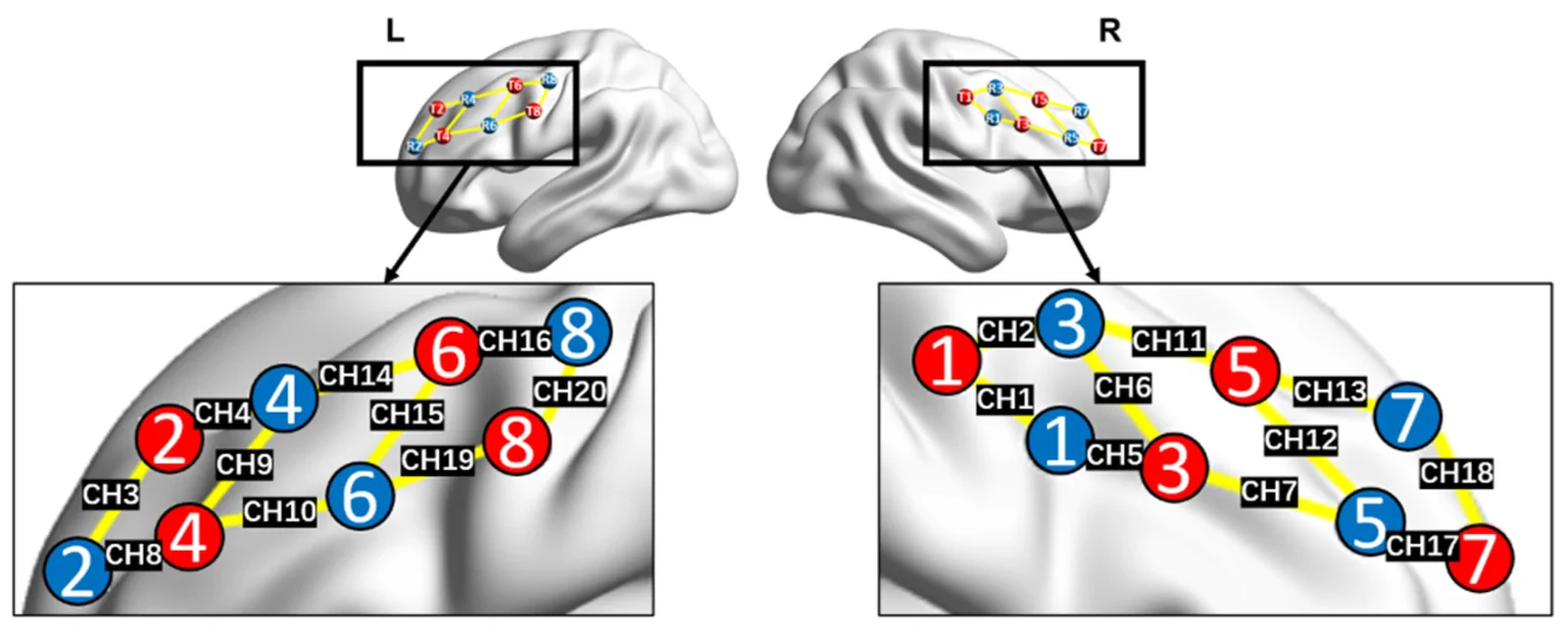
Arrangement of optodes and channels. The optode set in the LIGHTNIRS system consisted of eight transmitters (T) and eight receivers (R), forming 20 measurement channels. The numbers in red and blue circles indicate the transmitter and receiver IDs, respectively, while CH1-CH20 indicate the measurement channels.

Figure 3 shows the optode layout on the lateral scalp used in this study. A 3D digitizer (FASTRAK, Polhemus, Colchester, VT, USA) was used to record the coordinates of all optodes and anatomical landmarks. The channel coordinates were defined as the midpoints between the source and detector pairs. All real-space coordinates were registered to the Montreal Neurological Institute (MNI) space using the spatial registration function implemented in the NIRS-SPM toolbox (v4_r1) [35] in MATLAB (R2013b; MathWorks, Natick, MA, USA). Anatomical locations were reported based on the region with the highest probability estimated by NIRS-SPM. Because fNIRS has limited spatial resolution, these locations should be regarded as approximate and were used only as anatomical references in the discussion (Channel coordinates and anatomical assignments are provided in Supplementary Table S1).

#### 2.1.5. Behavioral Data Analysis

One-way repeated-measures analysis of variance (ANOVA) was conducted separately for the egg and corn soup conditions to analyze the saltiness and palatability ratings, with soup type (low-salt, low-salt + MPG + IMP, low-salt + soy sauce odor, low-salt + bacon odor, regular soup) as the within-subject factor. The Greenhouse–Geisser correction was applied to account for violations of sphericity. Post hoc comparisons were performed for significant main effects, with Tukey’s adjustment applied to all 10 pairwise comparisons. To evaluate the effectiveness of the saltiness enhancers, saltiness ratings for each low-salt condition containing an enhancer were compared with the low-salt and regular soup controls. Mean paired differences, Tukey-adjusted simultaneous 95% confidence intervals, and Cohen’s *d*_z_, a standardized measure of the magnitude of differences between paired conditions, were reported. In addition, supplementary linear mixed-effects models assessed serial-position effects within the three low-salt conditions containing saltiness enhancers (MPG + IMP, bacon odor, and soy sauce odor), separately for each soup category and rating outcome. Condition and serial position were included as fixed effects, with participant as a random intercept. The tests were adjusted using Holm’s procedure, and follow-up pairwise comparisons used Tukey’s adjustment. Statistical analyses were conducted in R (version 4.4.2) using the afex package (version 1.4-1) [36] for ANOVA and the emmeans package (version 1.11.1) [37] for post hoc comparisons, and the lme4 (version 1.1-37) [38] and lmerTest (version 3.1-3) packages [39] for the supplementary mixed-effects analyses.

The association between saltiness and palatability was examined separately for egg and corn soups using repeated-measures correlation, implemented with the rmcorr package [40] in R (version 4.4.2). This analysis accounted for repeated observations within participants and estimated the common within-participant linear association across conditions.

#### 2.1.6. Neural Data Analysis

##### Data Preprocessing and Hemodynamic Responses

Preprocessing, general linear model (GLM) analysis, and block averaging of hemodynamic responses were performed using Homer2 (version 2.8) [41], implemented in MATLAB (R2023a, MathWorks, Natick, MA, USA). Following data acquisition, raw Shimadzu-format text files were converted into a standard NIRS format based on the light intensity using a publicly available MATLAB script [42]. As the converted NIRS data lacked channel layout and trigger information, a source-detector file was created using the AtlasViewer function within Homer2 and subsequently incorporated into the NIRS file. Trigger information was added to the NIRS file in matrix form, enabling preprocessing and subsequent analyses using Homer2 [41].

The preprocessing pipeline was implemented using the functions provided in Homer2. In the processing stream, the optical intensity data were first normalized using the hmrIntensityNormalized function, which scales each channel based on its absolute mean value. The enPruneChannels function was then used to exclude low-quality channels, defined as those with a normalized intensity range (dRange) outside 0.5–2 and a signal-to-noise ratio below 2. Subsequently, the optical intensity signals from the retained channels were converted to optical density signals using the hmrIntensity2OD function. Motion artifacts were first detected using the hmrMotionArtifactByChannel function (tMotion = 0.5, tMask = 1.0, STDEVthresh = 10.0, AMPthresh = 5.0) and then corrected using the hmrMotionCorrectSplineSG function (*p* = 0.99, FrameSize_sec = 10). A bandpass filter (0.003–0.1 Hz) was applied to remove low-frequency drifts and attenuate high-frequency physiological and instrumental noise. Finally, the optical density data were converted to hemoglobin concentration changes using the hmrOD2Conc function. Block averaging was then performed with the hmrBlockAvg function, using a time window from 5 s before to 60 s after the onset of the sample-number cue, which served as the trigger onset, yielding preprocessed hemodynamic response time courses for each trial in each channel for each participant.

##### GLM Analysis

Hemodynamic responses were estimated using a deconvolution-based GLM with the Homer2 function hmrDeconvHRF_DriftSS. As defined in Equation (1), *Y*(*t*) denotes the observed fNIRS signal at time *t* as follows:

(1) $$Y\left(t\right)=\sum_{k=1}^{10}\left[\beta_{k,1}X_{k,1}\left(t\right)+\beta_{k,2}X_{k,2}\left(t\right)\right]+\gamma_{0}+\gamma_{1}t+\epsilon \left(t\right)$$

where the index *k* refers to the experimental condition (*k* = 1, …, 10). *X_k,_*_1_ and *X_k,_*_2_ represent the regressors for each condition and capture the temporal dynamics of the hemodynamic response. The coefficients *β_k,_*_1_ and *β_k,_*_2_ quantify the amplitude and temporal shifts of the response, respectively. The terms *γ*_0_ and *γ*_1_*t* model the baseline and linear drift, respectively, and *ϵ*(*t*) represents the residual error. The GLM parameters were estimated using ordinary least squares based on the fNIRS-GLM analysis framework described by Ye et al. [35].

Regressors *X_k,_*_1_ and *X_k,_*_2_ were constructed by convolving the stimulus time series *s_k_* with the basis functions *h*_1_ and *h*_2_ (Equation (2)), which were defined using a modified gamma function *g* and its temporal derivative *g′*, each convolved with a square wave (*b*(*t*) = 1, 0 ≤ *t* ≤ T) of duration T (16 s), to model the hemodynamic response function HRF (Equation (3)).

*X_k_*_,1_(*t*) = (*s_k_* ∗ *h*_1_)(*t*), *X_k_*_,2_(*t*) = (*s_k_* ∗ *h*_2_)(*t*) (2)

*h*_1_(*t*) = (*g* ∗ *b*)(*t*), *h*_2_(*t*) = (*g′* ∗ *b*)(*t*) (3)

The parameters of the gamma function (Equation (4)) were set to *τ* = 8 and *σ* = 10, which were chosen to approximate the typical shape of the hemodynamic response in the present study.

(4) $$g\left(t;\;\tau ,\;\sigma \right)=\frac{{\left(t-\tau \right)}^{2}}{\sigma^{2}}\exp\left(1-\frac{{\left(t-\tau \right)}^{2}}{\sigma^{2}}\right)$$

The coefficient of the gamma-based canonical HRF (*β*_1_), which reflects the amplitude of the hemodynamic response, was used as the response amplitude measure for each condition across all participants and channels. For each channel, a separate one-way repeated-measures ANOVA was conducted for egg and corn soups, with soup type as a within-subject factor to examine whether the response amplitudes differed across the five conditions. Oxygenated hemoglobin (HbO) was used for the primary analysis, and the same analyses were performed on deoxygenated hemoglobin (HbR) as an exploratory robustness check. Greenhouse–Geisser corrections were applied to account for potential violations of sphericity. Within each soup category, the resulting *p*-values for the effect were adjusted across all 20 channels using the Benjamini–Hochberg procedure to control the false discovery rate. Both *p*-values before cross-channel adjustment and BH-adjusted *p*-values are reported. Statistical significance was defined as a BH-adjusted *p*-value < 0.05. As a post hoc exploratory reporting criterion, channels with *p*-values < 0.01 before cross-channel adjustment that did not survive BH correction were also described, alongside their BH-adjusted *p*-values. These exploratory findings were not considered statistically significant. Post hoc pairwise comparisons were performed for the channels identified by either of the above criteria, with Tukey’s adjustment applied to the 10 pairwise comparisons within each channel. Similarly, supplementary mixed-effects models assessed serial-position effects within the three enhancer conditions in channels showing significant main effects, with condition and position as fixed effects and participant as a random intercept.

### 2.2. Experiment 2

#### 2.2.1. Participants

Thirty Japanese university students (14 males and 16 females; *M*_age_ = 22.07, *SD* = 1.34) participated in Experiment 2. A priori power analysis was conducted using G*Power 3.1.9.7. Assuming an effect size of *f* = 0.25, α = 0.05, and a power of 0.80, the required sample size was estimated to be 28 participants. A total of 30 participants were ultimately recruited. As in Experiment 1, all participants reported good health with normal taste and olfactory functions. The pre-experimental instructions and informed consent procedures were the same as those described in Experiment 1. The study protocol was also approved by the Ethics Committee of the Kawauchi Minami Campus, Tohoku University (approval no. [2023-010]).

#### 2.2.2. Stimuli and Experimental Design

The taste stimuli in Experiment 2 consisted of regular and low-salt egg and corn soups used in Experiment 1. Visual stimuli were manipulated using three packaging cues: regular packaging, low-salt packaging, and no packaging as the control condition. The regular and low-salt soups were prepared using the same procedures as in Experiment 1 (Table 1).

Because the purpose of this experiment was not to compare egg and corn soups, subsequent analyses were conducted only within each soup category. Specifically, for both egg and corn soups, a 2 × 3 within-subject design was used, incorporating two factors: soup type (regular and low-salt) and package (regular packaging, low-salt packaging, and no packaging).

#### 2.2.3. Procedure

**Figure 4:**
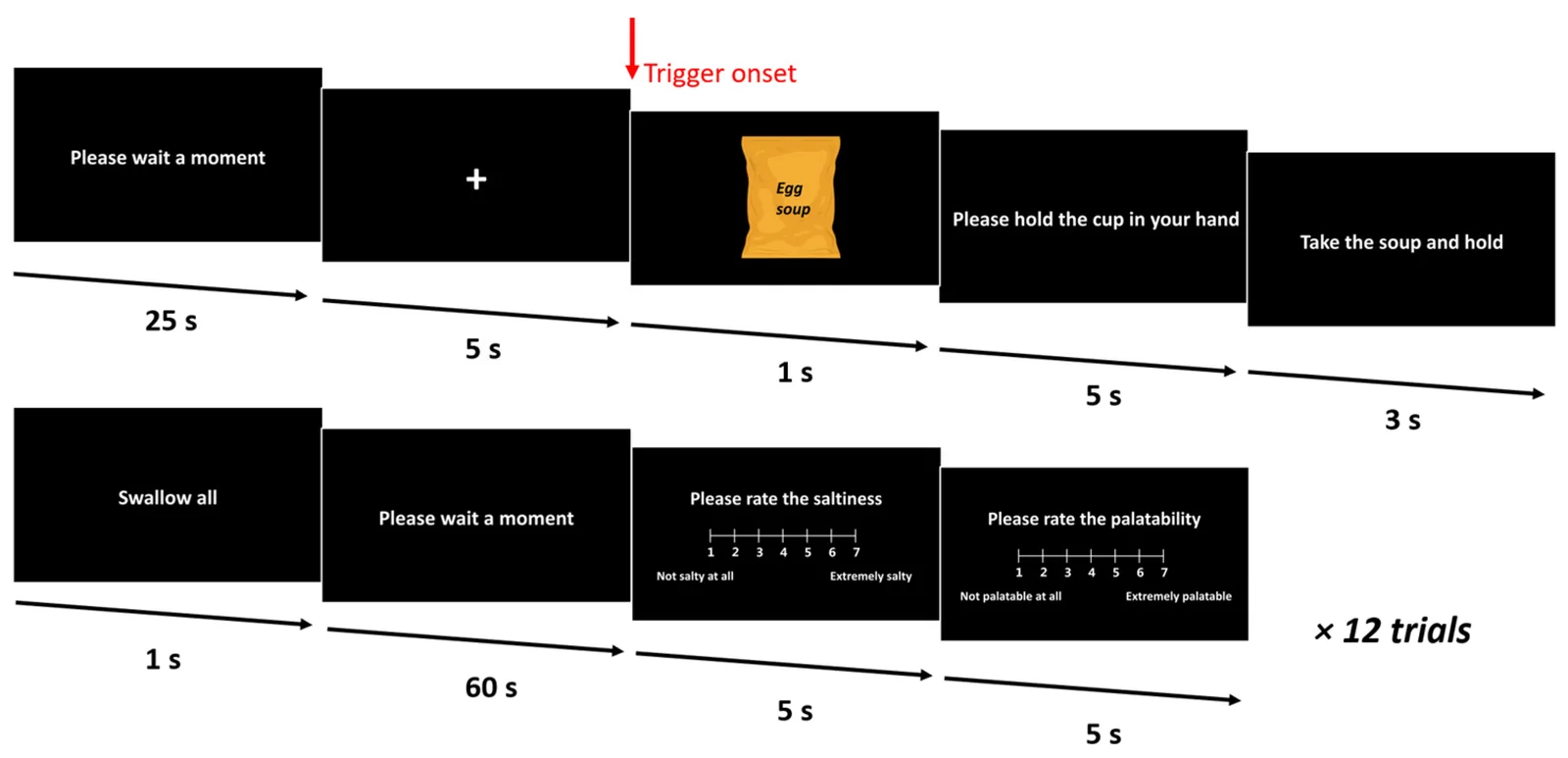
Flow chart of the procedure for Experiment 2.

The procedure in Experiment 2 (Figure 4) followed that in Experiment 1, except that the sample-number cue was replaced by a packaging cue presented for 1 s and no separate cup-replacement period was included. Participants then picked up the cup and held it for 5 s while preparing to drink, took some soup into their mouths, and held it there for 3 s before receiving a swallowing instruction for 1 s. Following the 60 s recording period, they rated the saltiness and palatability of the soup using the same 7-point Likert scales as in Experiment 1. The experiment comprised 12 conditions, each of which was presented once. The two soup categories (egg and corn) were presented alternately across trials. The starting soup category was counterbalanced across participants. Within each soup category, each of the six conditions was presented once in a randomized order.

#### 2.2.4. fNIRS Data Acquisition and Channel Localization

**Figure 5:**
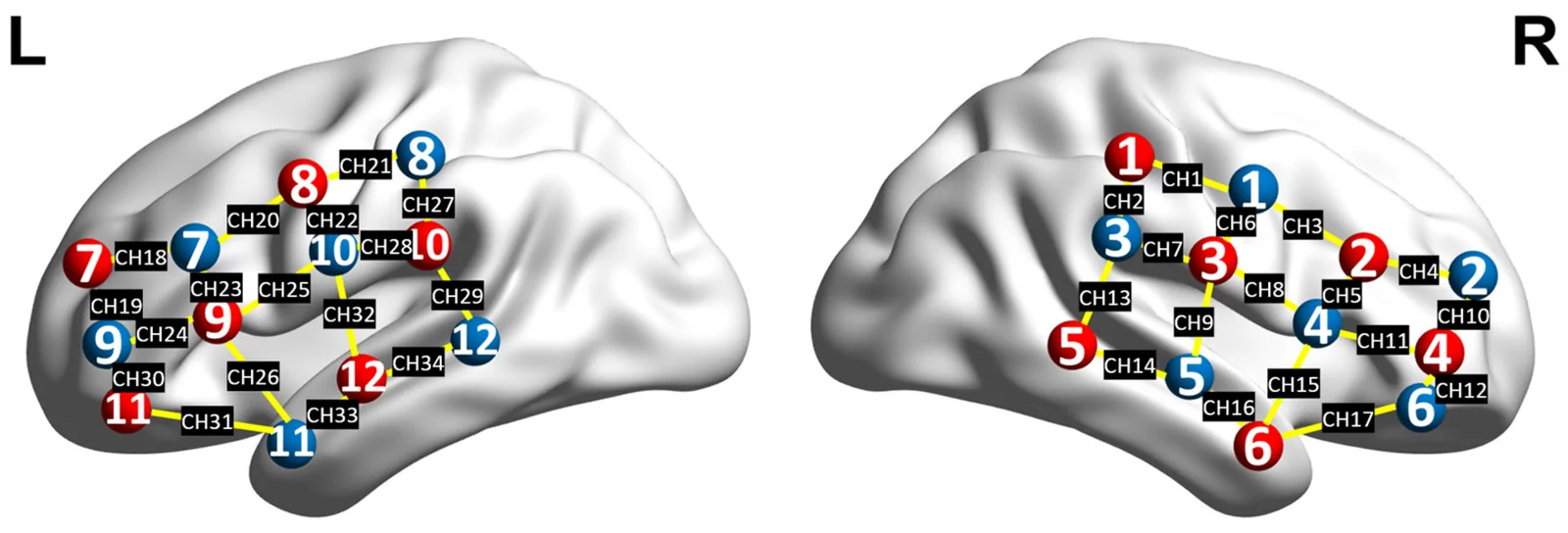
Optode set in the FOIRE-3000 system consisting of 12 transmitters (T) and 12 receivers (R), forming 34 measurement channels. The numbers in red and blue circles indicate the transmitter and receiver IDs, respectively, while CH1-CH34 indicate the measurement channels.

Hemodynamic data were recorded using a FOIRE-3000 system (Shimadzu Corporation, Kyoto, Japan), which provided a greater number of measurement channels than the system used in Experiment 1. The system consisted of 12 pairs of probes and yielded data from 34 channels. Data were recorded at a sampling rate of 10 Hz using three NIR wavelengths: 780, 805, and 830 nm. Short-separation channels were not available. Figure 5 shows the optode layout over the lateral scalp used in this study. The procedures for coordinate acquisition and spatial registration to MNI space were the same as those used in Experiment 1 (Channel coordinates and anatomical assignments are provided in Supplementary Table S2).

#### 2.2.5. Behavioral Data Analysis

A 2 × 3 repeated-measures ANOVA was conducted separately on saltiness and palatability ratings for each soup category, with soup type (regular, low-salt) and package (regular packaging, low-salt packaging, no packaging) as the within-subject factors. The Greenhouse–Geisser correction was applied to account for violations of sphericity. Post hoc pairwise comparisons were performed for significant main effects. For significant interactions, package conditions were compared within each soup type, and soup types were compared within each package condition. Tukey’s adjustment was applied within each set of pairwise comparisons. In addition, serial-position effects were assessed using supplementary linear mixed-effects models fitted separately for each soup category and rating outcome. Soup type, package, their interaction, serial position, and whether the soup category was presented first were included as fixed effects, with participant included as a random intercept. Serial position was numbered from 1 to 6 separately for egg and corn soups, according to the order in which each was presented. Holm’s procedure was applied across the four tests of serial-position effects. All analyses were conducted in R (version 4.4.2) using the same packages as in Experiment 1: afex (version 1.4-1) [36] for ANOVA, emmeans (version 1.11.1) [37] for post hoc comparisons, and lme4 (version 1.1-37) [38] and lmerTest (version 3.1-3) [39] for the supplementary mixed-effects analyses.

The association between saltiness and palatability was examined using the same repeated-measures correlation procedure as in Experiment 1, separately for egg and corn soups.

#### 2.2.6. Neural Data Analysis

Data pre-processing and GLM-based analyses were performed using the same procedures as those described in Experiment 1. The only difference from Experiment 1 was the parameter settings used for the GLM. The gamma function parameters were kept unchanged at τ = 8 and σ = 10, but the function was convolved with a square wave of *T* = 10 s to account for the shorter task period following the trigger.

For each channel, the same 2 × 3 repeated-measures ANOVA with soup type and package included as within-subject factors was conducted separately for the egg and corn soup conditions to examine their main effects and interactions on response amplitude. The same analyses were also performed on HbR as an exploratory robustness check. Greenhouse–Geisser corrections were applied where appropriate. For each effect, *p*-values were adjusted across all 34 channels separately within each soup category using the Benjamini–Hochberg procedure. Both *p*-values before cross-channel adjustment and BH-adjusted *p*-values are reported. The same criteria for statistical significance and exploratory reporting as in Experiment 1 were applied. Serial-position effects in channels showing significant main effects were additionally assessed using the supplementary mixed-effects modeling approach described for the behavioral data.

In Experiment 1, only E1-CH19 survived Benjamini–Hochberg correction. This channel was approximately localized to the left dorsolateral prefrontal cortex (DLPFC; estimated MNI coordinates: −59, 9, 34), with 67.77% overlap with BA9 and 32.23% with BA6. Motivated by this finding, we examined responses in Experiment 2 at channels approximately localized to the bilateral DLPFC: E2-CH03 (MNI: 65, 7, 30; 40% overlap with BA9) and E2-CH20 (MNI: −54, 24, 31; 53.98% overlap with BA9 and 31.83% with BA46). Given the inherent spatial limitations of fNIRS-based anatomical localization and the differences in probe geometry and acquisition systems, these channels were selected to examine responses within the broader DLPFC region rather than to establish precise spatial correspondence with E1-CH19.

## 3. Results

### 3.1. Experiment 1

#### 3.1.1. Behavioral Results

##### Saltiness Rating

For both egg and corn soups, one-way repeated-measures ANOVAs revealed significant main effects of soup type on perceived saltiness (egg soup: *F*(3.77, 109.32) = 7.74, *MSE* = 0.75, *p* < 0.001, η^2^_p_ = 0.21; corn soup: *F*(2.98, 86.30) = 15.45, *MSE* = 1.22, *p* < 0.001, η^2^_p_ = 0.35).

**Figure 6:**
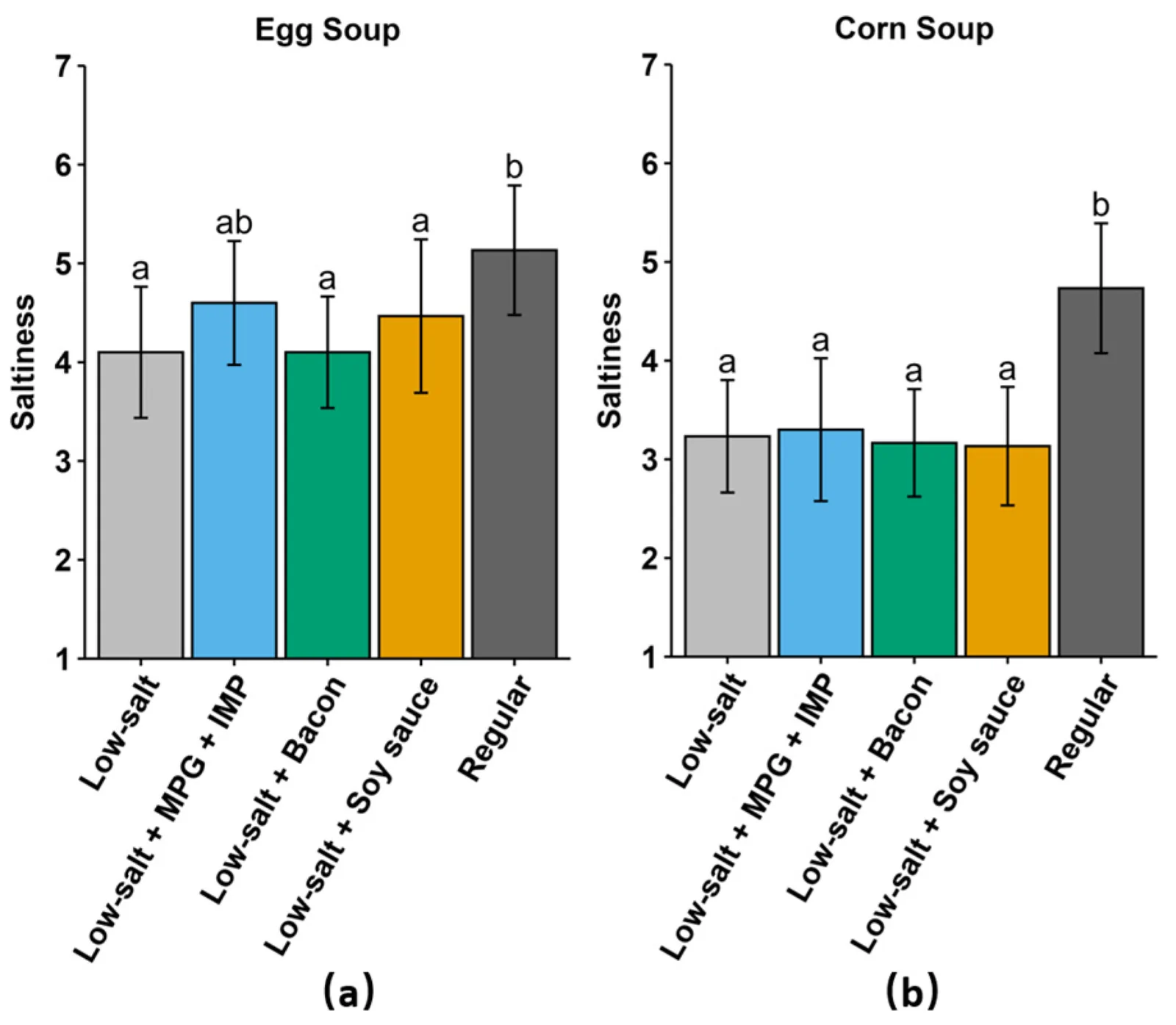
Saltiness ratings across the five soup types for egg (**a**) and corn (**b**) soup. The colored bars represent the mean saltiness ratings, and the error bars indicate the 95% confidence intervals. Bars that do not share lowercase letters differ significantly within each soup category.

For egg soup, Tukey-adjusted post hoc comparisons (Figure 6a) showed that regular soup was rated significantly saltier than low-salt soup (adjusted *p* < 0.001), low-salt soup with bacon odor (adjusted *p* < 0.001), and low-salt soup with soy sauce odor (adjusted *p* < 0.05), but not low-salt soup with MPG + IMP (adjusted *p* > 0.05). The estimated differences for the enhancer conditions relative to regular soup were −0.53 points for MPG + IMP (Cohen’s *d*_z_ = −0.45, 95% CI [−1.17, 0.10]), −1.03 points for bacon odor (Cohen’s *d*_z_ = −1.00, 95% CI [−1.58, −0.48]), and −0.67 points for soy sauce odor (Cohen’s *d*_z_ = −0.56, 95% CI [−1.30, −0.04]). None of the three enhancer conditions differed significantly from the low-salt control. The estimated differences relative to low-salt soup were 0.50 points for MPG + IMP (Cohen’s *d*_z_ = 0.42, 95% CI [−0.13, 1.13]), 0.00 points for bacon odor (Cohen’s *d*_z_ = 0.00, 95% CI [−0.64, 0.64]), and 0.37 points for soy sauce odor (Cohen’s *d*_z_ = 0.32, 95% CI [−0.23, 0.97]; all adjusted *p* > 0.05). Supplementary analyses revealed no significant serial-position effects (*F*(2, 56) = 0.41, Holm-adjusted *p* = 1.000).

For corn soup, Tukey-adjusted post hoc comparisons (Figure 6b) showed that regular soup was rated significantly saltier than low-salt soup (adjusted *p* < 0.001), low-salt soup with MPG + IMP (adjusted *p* < 0.01), low-salt soup with bacon odor (adjusted *p* < 0.001), and low-salt soup with soy sauce odor (adjusted *p* < 0.001). The estimated differences for the enhancer conditions relative to regular soup were −1.43 points for MPG + IMP (Cohen’s *d*_z_ = −0.80, 95% CI [−2.39, −0.48]), −1.57 points for bacon odor (Cohen’s *d*_z_ = −1.23, 95% CI [−2.24, −0.89]), and −1.60 points for soy sauce odor (Cohen’s *d*_z_ = −1.41, 95% CI [−2.20, −1.00]). None of the three enhancer conditions differed significantly from the low-salt control. The estimated differences relative to low-salt soup were 0.07 points for MPG + IMP (Cohen’s *d*_z_ = 0.05, 95% CI [−0.59, 0.72]), −0.07 points for bacon odor (Cohen’s *d*_z_ = −0.06, 95% CI [−0.67, 0.54]), and −0.10 points for soy sauce odor (Cohen’s *d*_z_ = −0.08, 95% CI [−0.74, 0.54]; all adjusted *p* > 0.05). No significant differences were observed among the four low-salt corn soup conditions. Supplementary analyses revealed a significant serial-position effect (*F*(2, 56) = 4.86, Holm-adjusted *p* < 0.05). Follow-up comparisons showed higher saltiness ratings at the fourth than at the second position within the corn soup block (Tukey-adjusted *p* < 0.05).

##### Palatability Rating

**Figure 7:**
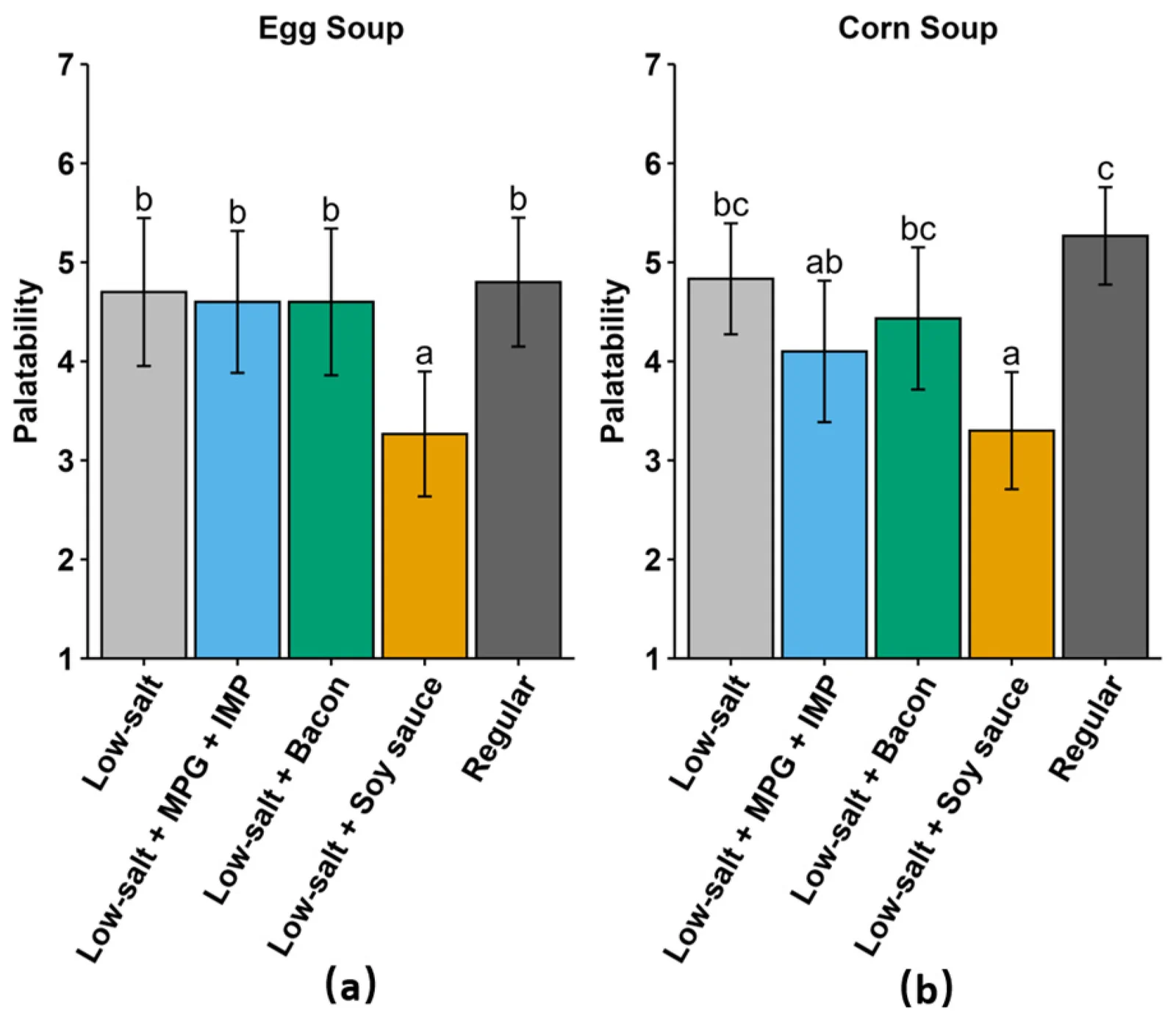
Palatability ratings across the five soup types for egg (**a**) and corn (**b**) soups. The colored bars represent the mean palatability ratings, and the error bars indicate the 95% confidence intervals. Bars that do not share lowercase letters differ significantly within each soup category.

Significant main effects of soup type on perceived palatability were observed for both egg and corn soups (egg soup: *F*(3.15, 91.30) = 11.40, *MSE* = 1.35, *p* < 0.001, η^2^_p_ = 0.28; corn soup: *F*(3.66, 106.14) = 14.67, *MSE* = 1.25, *p* < 0.001, η^2^_p_ = 0.34). For egg soup, post hoc comparisons (Figure 7a) show that the low-salt soup with soy sauce odor was rated significantly less palatable than the low-salt soup (adjusted *p* < 0.001), low-salt soup with MPG + IMP (adjusted *p* < 0.001), low-salt soup with bacon odor (adjusted *p* < 0.001), and regular soup (*p* < 0.001). For corn soup, post hoc comparisons (Figure 7b) show that regular soup was rated significantly more palatable than low-salt soup with MPG + IMP (adjusted *p* < 0.001) or low-salt soup with soy sauce odor (adjusted *p* < 0.001). In addition, the low-salt soup with soy sauce odor was rated as significantly less palatable than the low-salt soup (adjusted *p* < 0.001) and the low-salt soup with bacon odor (adjusted *p* < 0.01). No significant serial-position effect on palatability was observed for either egg soup (*F*(2, 56) = 1.11, Holm-adjusted *p* > 0.05) or corn soup (*F*(2, 56) = 0.78, Holm-adjusted *p* > 0.05).

Repeated-measures correlation revealed no significant within-participant association between perceived saltiness and palatability for egg soup (*r*_rm_(119) = 0.165, 95% CI [−0.014, 0.333], *p* = 0.071). For corn soup, a significant positive within-participant association was observed (*r*_rm_(119) = 0.310, 95% CI [0.139, 0.463], *p* < 0.001).

#### 3.1.2. fNIRS Results

Following quality control, data from participant 7 were excluded across all conditions for E1-CH13. This left 29 participants for E1-CH13 and 30 for all remaining channels.

The results of HbO showed a significant effect of soup type at E1-CH19 for egg soup (*F*(3.13, 90.82) = 5.43, *p* < 0.01, BH-adjusted *p* < 0.05, η^2^_p_ = 0.158). Additionally, an exploratory effect was observed at E1-CH16 (*F*(3.36, 97.57) = 3.80, *p* < 0.01, BH-adjusted *p* = 0.098, η^2^_p_ = 0.116), which did not survive BH correction. Neither channel showed a significant effect in the supplementary HbR robustness analysis that survived BH correction or met the exploratory reporting criterion. For corn soup, no channel in the HbO analysis survived BH correction or met the exploratory reporting criterion. Supplementary linear mixed-effects analyses detected no significant serial-position effects within positions 2–4 for egg soup at either E1-CH19 (*F*(2, 56) = 1.20, Holm-adjusted *p* = 0.619) or E1-CH16 (*F*(2, 56) = 0.10, Holm-adjusted *p* = 0.902). Channel-wise HbO beta estimates and response time courses are shown in Supplementary Figures S2–S3 and S4–S5, respectively. Complete HbO and HbR results are provided in Supplementary Table S3a,b.

**Figure 8:**
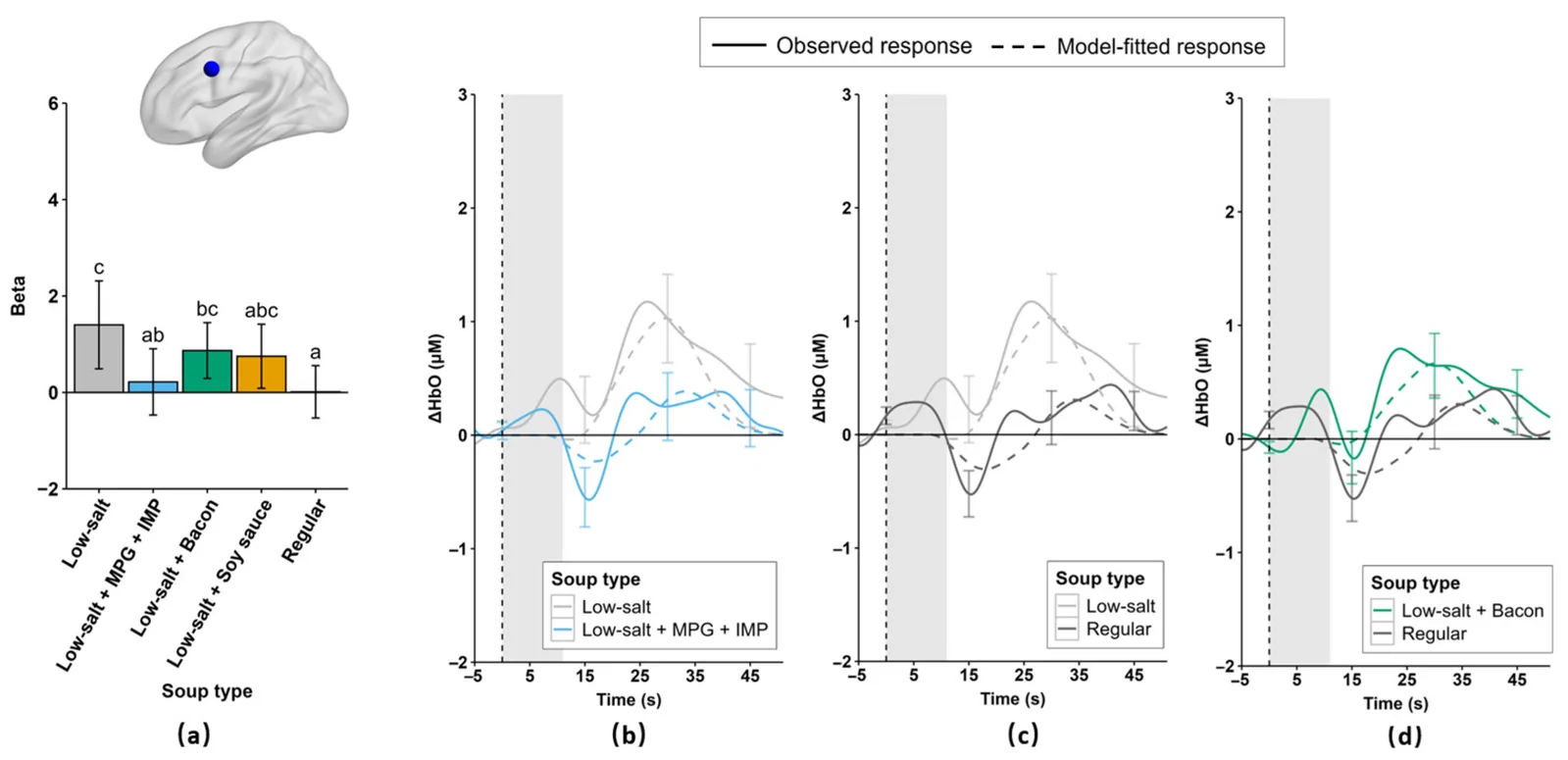
Neural response characteristics of E1-CH19 for egg soup. (**a**) Cortical location of E1-CH19 and the magnitude of its hemodynamic responses across all five soup types. The blue dot indicates the approximate cortical location of E1-CH19. The bar graph represents the mean beta estimates for each soup type, and the error bars indicate the 95% confidence intervals. Lowercase letters above the bars summarize Tukey-adjusted post hoc comparisons; bars that do not share the same letter differ significantly (adjusted *p* < 0.05). In (**b**–**d**), the time-course HbO responses are shown for the soup type pairs that differed significantly: (**b**) low-salt versus low-salt + MPG + IMP, (**c**) low-salt versus regular, and (**d**) low-salt + bacon odor versus regular. Solid lines represent the preprocessed block-averaged changes in HbO concentration, and dashed lines represent the model-fitted HbO responses estimated using the GLM with a modified gamma basis function. The same panel organization and plotting conventions are used for all subsequent neural response figures.

For E1-CH19 (Figure 8a), post hoc analysis showed that the low-salt soup elicited significantly higher responses than both the low-salt soup with MPG + IMP (*t*(29) = 3.55, adjusted *p* < 0.05, 95% CI [0.216, 2.150]; Figure 8b) and the regular soup (*t*(29) = 3.37, adjusted *p* < 0.05, 95% CI [0.189, 2.588]; Figure 8c). In addition, the low-salt soup with bacon odor elicited significantly higher responses than the regular soup (*t*(29) = 3.21, adjusted *p* < 0.05, 95% CI [0.080, 1.633]; Figure 8d).

**Figure 9:**
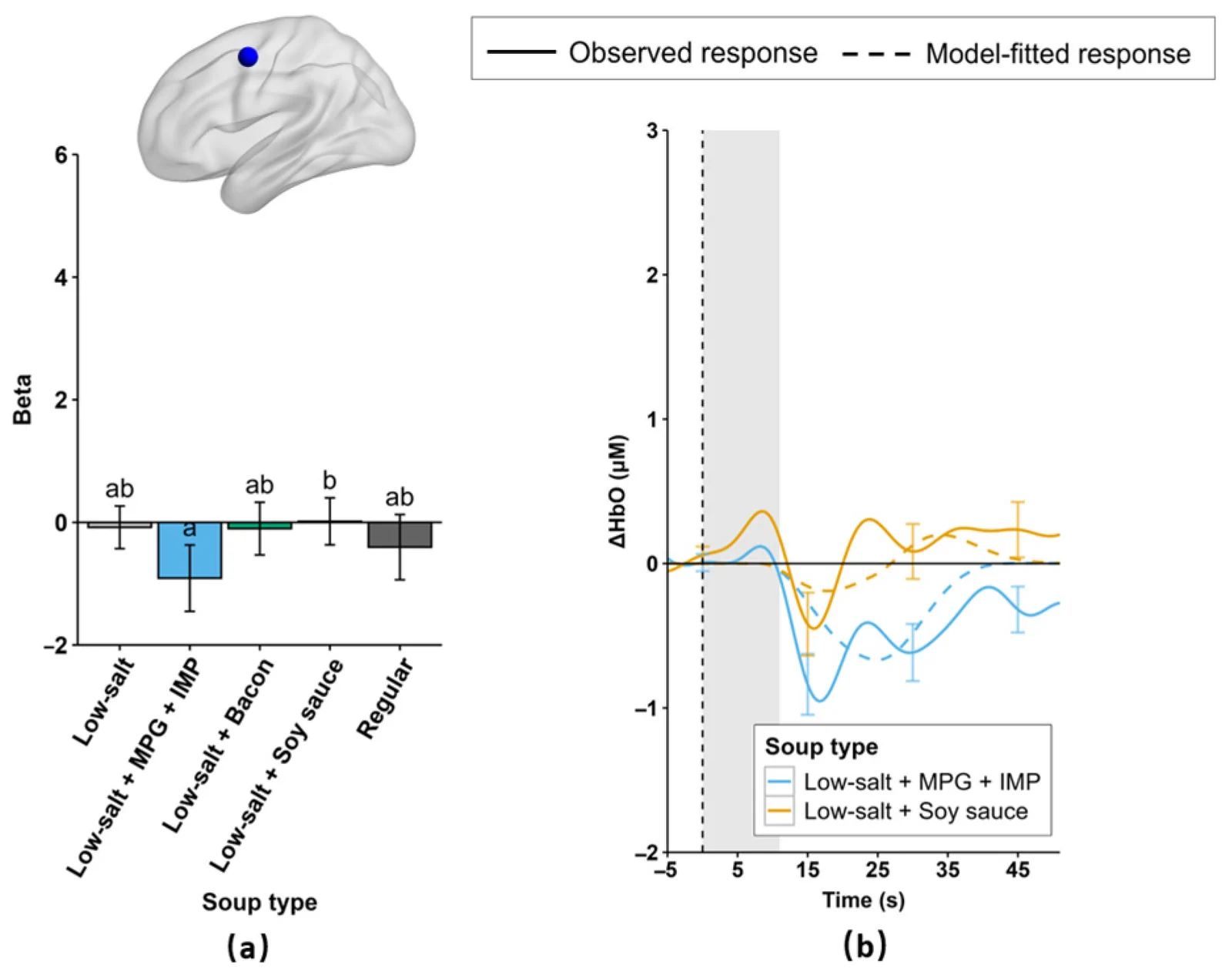
Neural response characteristics of E1-CH16 for egg soup. (**a**) Cortical location of E1-CH16 and mean beta estimates across all soup types. The blue dot indicates the approximate cortical location of E1-CH16. Different lowercase letters above the bars indicate statistically significant differences between conditions (adjusted *p* < 0.05). (**b**) Time-course HbO responses for low-salt + MPG + IMP versus low-salt + soy sauce odor.

For E1-CH16 (Figure 9a), post hoc comparisons showed that low-salt soup with MPG + IMP elicited lower response amplitudes than low-salt soup with soy sauce odor (*t*(29) = −3.36, adjusted *p* < 0.05, 95% CI [−1.728, −0.124]; Figure 9b). The remaining pairwise comparisons were not significant (all *p* > 0.05). This finding was considered exploratory because the channel-level condition effect did not survive BH correction.

#### 3.1.3. Hemodynamic Response–Behavioral Correlation Analysis

Repeated-measures correlations were used to examine the associations between neural responses and subjective saltiness and palatability ratings, focusing on the channels reported above. Correlations were calculated for all 20 channels that passed quality control, with BH correction applied jointly across channels and both behavioral measures within each soup category. No correlations survived correction for either egg soup or corn soup (Complete correlation results are provided in Supplementary Table S5).

### 3.2. Experiment 2

#### 3.2.1. Behavioral Results

##### Saltiness Rating

**Figure 10:**
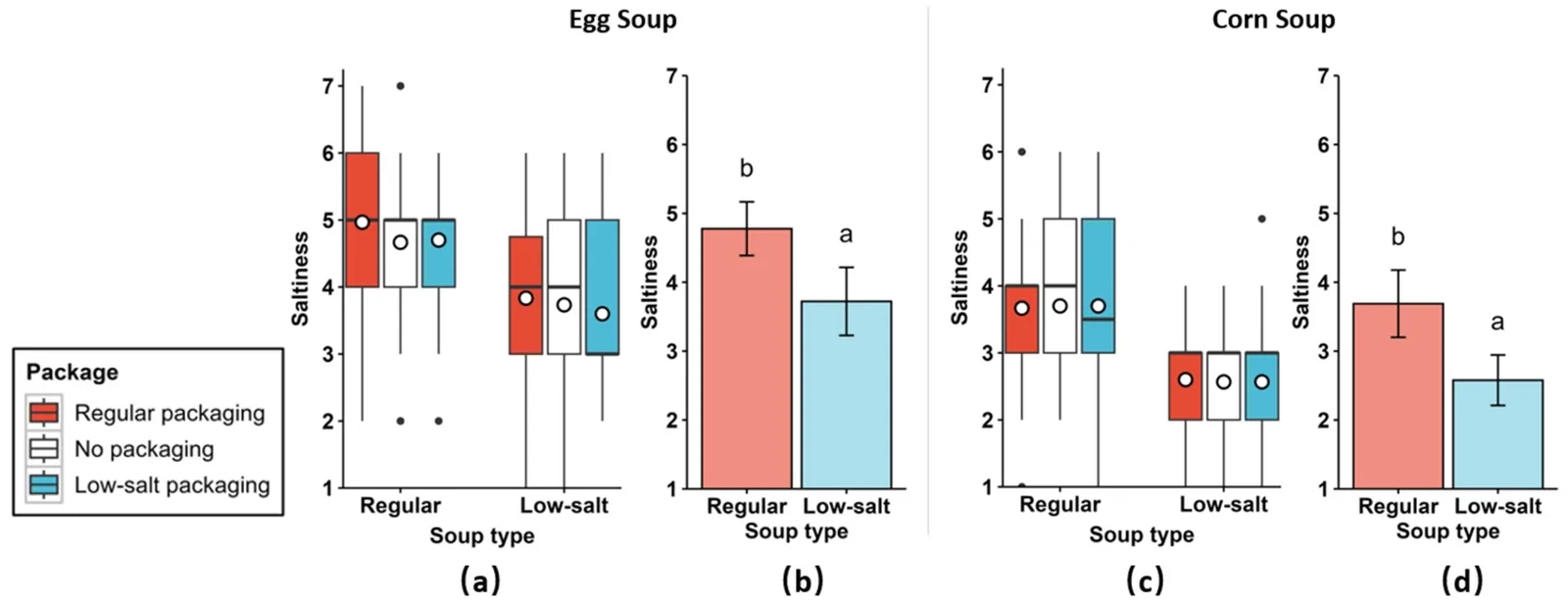
Saltiness ratings for egg and corn soups in Experiment 2. (**a**,**c**) Distribution of saltiness ratings across soup types and package conditions for egg and corn soups, respectively. (**b**,**d**) Mean saltiness ratings for regular and low-salt egg and corn soups, respectively, collapsed across package conditions. Error bars indicate the 95% confidence intervals, and different lowercase letters indicate significant differences between soup types.

The saltiness ratings for egg and corn soups are shown in Figure 10a and Figure 10c, respectively. No significant soup type × package interaction was observed in either soup category (egg soup: *F*(1.90, 55.09) = 0.22, *MSE* = 0.83, *p* = 0.79, η^2^_p_ = 0.01; corn soup: *F*(1.92, 55.69) = 0.05, *MSE* = 0.47, *p* = 0.95, η^2^_p_ = 0.002). There was also no significant main effect of package for either soup category (egg soup: *F*(1.88, 54.50) = 2.13, *MSE* = 0.52, *p* = 0.13, η^2^_p_ = 0.07; corn soup: *F*(1.81, 52.51) = 0.00, *MSE* = 0.48, *p* = 1.00, η^2^_p_ = 0.00). However, a significant main effect of soup type was found for both egg and corn soups (egg soup: *F*(1, 29) = 38.92, *MSE* = 1.29, *p* < 0.001, η^2^_p_ = 0.57; corn soup: *F*(1, 29) = 54.72, *MSE* = 1.02, *p* < 0.001, η^2^_p_ = 0.65). Post hoc pairwise comparisons (Figure 10b,d) showed that regular soup was rated significantly saltier than low-salt soup for both soups (*p* < 0.001). Supplementary analyses revealed no significant serial-position effects for either egg soup (*F*(5, 140) = 1.27, Holm-adjusted *p* > 0.05) or corn soup (*F*(5, 140) = 0.48, Holm-adjusted *p* > 0.05).

##### Palatability Rating

**Figure 11:**
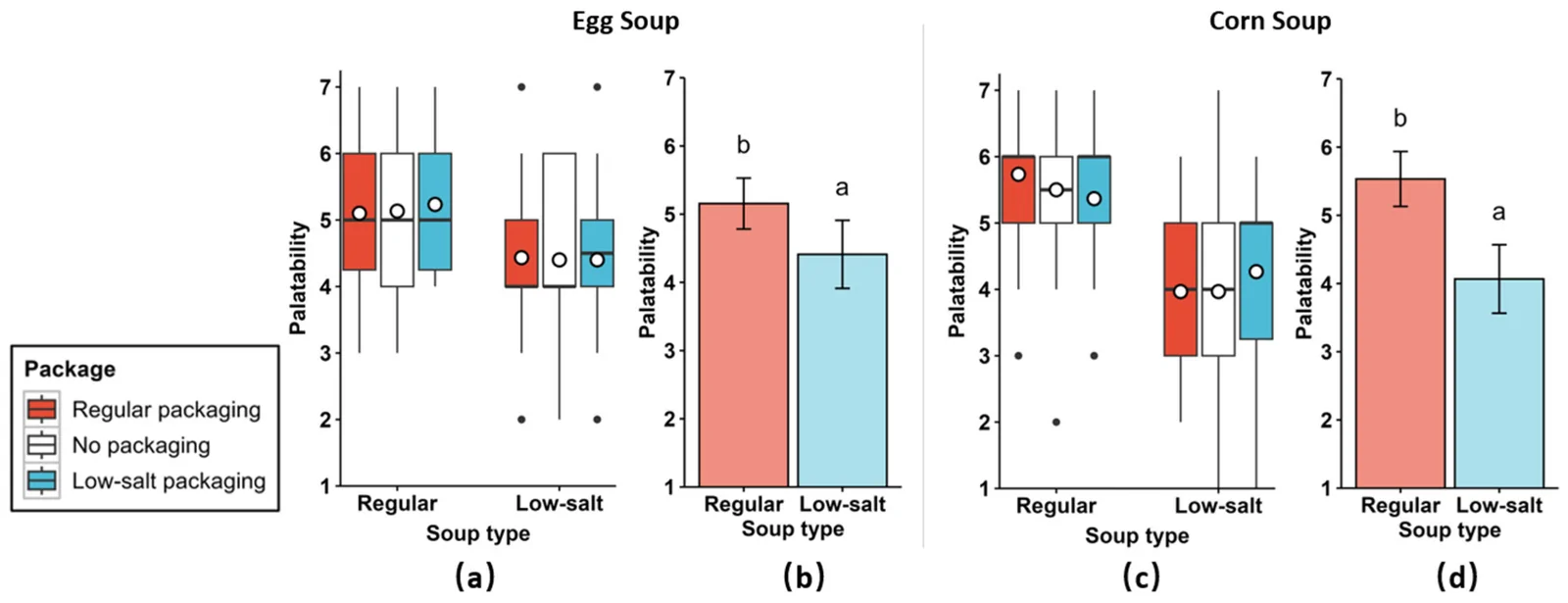
Palatability ratings for egg and corn soups in Experiment 2. (**a**,**c**) Distribution of palatability ratings across soup type and package conditions for egg and corn soups. (**b**,**d**) Mean palatability ratings for regular and low-salt egg and corn soups. Error bars indicate the 95% confidence intervals, and different lowercase letters indicate significant differences between soup types.

The palatability ratings for egg and corn soups are shown in Figure 11a and Figure 11c, respectively. No significant soup type × package interaction was observed in either soup category (egg soup: *F*(1.95, 56.66) = 0.25, *MSE* = 0.43, *p* = 0.77, η^2^_p_ = 0.01; corn soup: *F*(1.69, 49.12) = 2.09, *MSE* = 0.97, *p* = 0.14, η^2^_p_ = 0.07). There was also no significant main effect of package for either soup type (egg soup: *F*(1.81, 52.60) = 0.10, *MSE* = 0.56, *p* = 0.89, η^2^_p_ = 0.003; corn soup: *F*(1.94, 56.15) = 0.43, *MSE* = 0.52, *p* = 0.65, η^2^_p_ = 0.01). However, a significant main effect of soup type was found for both egg and corn soups (egg soup: *F*(1, 29) = 19.96, *MSE* = 1.25, *p* < 0.001, η^2^_p_ = 0.41; corn soup: *F*(1, 29) = 57.06, *MSE* = 1.70, *p* < 0.001, η^2^_p_ = 0.66). Post hoc pairwise comparisons (Figure 11b,d) showed that regular soup was rated significantly more palatable than the low-salt soup for both soups (*p* < 0.01). Serial-position effects on palatability were also not significant for egg soup (*F*(5, 140) = 0.69, Holm-adjusted *p* > 0.05) or corn soup (*F*(5, 140) = 1.73, Holm-adjusted *p* > 0.05).

Repeated-measures correlation revealed significant positive within-participant associations between perceived saltiness and palatability for both egg soup (*r*_rm_(149) = 0.448, 95% CI [0.311, 0.568], *p* < 0.001) and corn soup (*r*_rm_(149) = 0.655, 95% CI [0.553, 0.738], *p* < 0.001).

#### 3.2.2. fNIRS Results

##### Results Across All Channels

Following quality control, data were excluded across all conditions for the following channels and participants: E2-CH3 (participant 27), E2-CH9 (participant 4), E2-CH13 (participant 19), E2-CH14 (participants 4 and 19), E2-CH16 (participant 4), and E2-CH18, E2-CH23, and E2-CH27 (participant 5). This left 28 participants for E2-CH14, 29 for the other seven affected channels, and 30 for all remaining channels.

The HbO results showed no significant main effects or interactions after BH correction in either soup category. For egg soup, an exploratory soup type × package interaction was observed at E2-CH6 (*F*(1.67, 48.55) = 5.63, *p* < 0.01, BH-adjusted *p* = 0.29, η^2^_p_ = 0.163). Additionally, an exploratory main effect of soup type was observed at E2-CH32 (*F*(1, 29) = 11.66, *p* < 0.01, BH-adjusted *p* = 0.06, η^2^_p_ = 0.287). For corn soup, an exploratory soup type × package interaction was observed at E2-CH17 (*F*(1.79, 51.86) = 5.94, *p* < 0.01, BH-adjusted *p* = 0.21, η^2^_p_ = 0.170). Of these three effects, only the soup type × package interaction at E2-CH17 was significant in the supplementary HbR robustness analysis after BH correction (*F*(1.76, 50.92) = 9.37, *p* < 0.001, BH-adjusted *p* = 0.0197). Supplementary analyses showed no significant serial-position effects for egg soup at E2-CH6 (*F*(5, 140) = 1.99, Holm-adjusted *p* = 0.168) or E2-CH32 (*F*(5, 140) = 0.27, Holm-adjusted *p* = 0.927). For corn soup, the serial-position effect at E2-CH17 did not survive Holm correction (*F*(5, 140) = 2.44, *p* = 0.037, Holm-adjusted *p* = 0.111). Channel-wise HbO beta estimates and response time courses are shown in Supplementary Figures S6–S7 and S8–S9, respectively. Complete HbO and HbR results are provided in Supplementary Table S4a,b.

**Figure 12:**
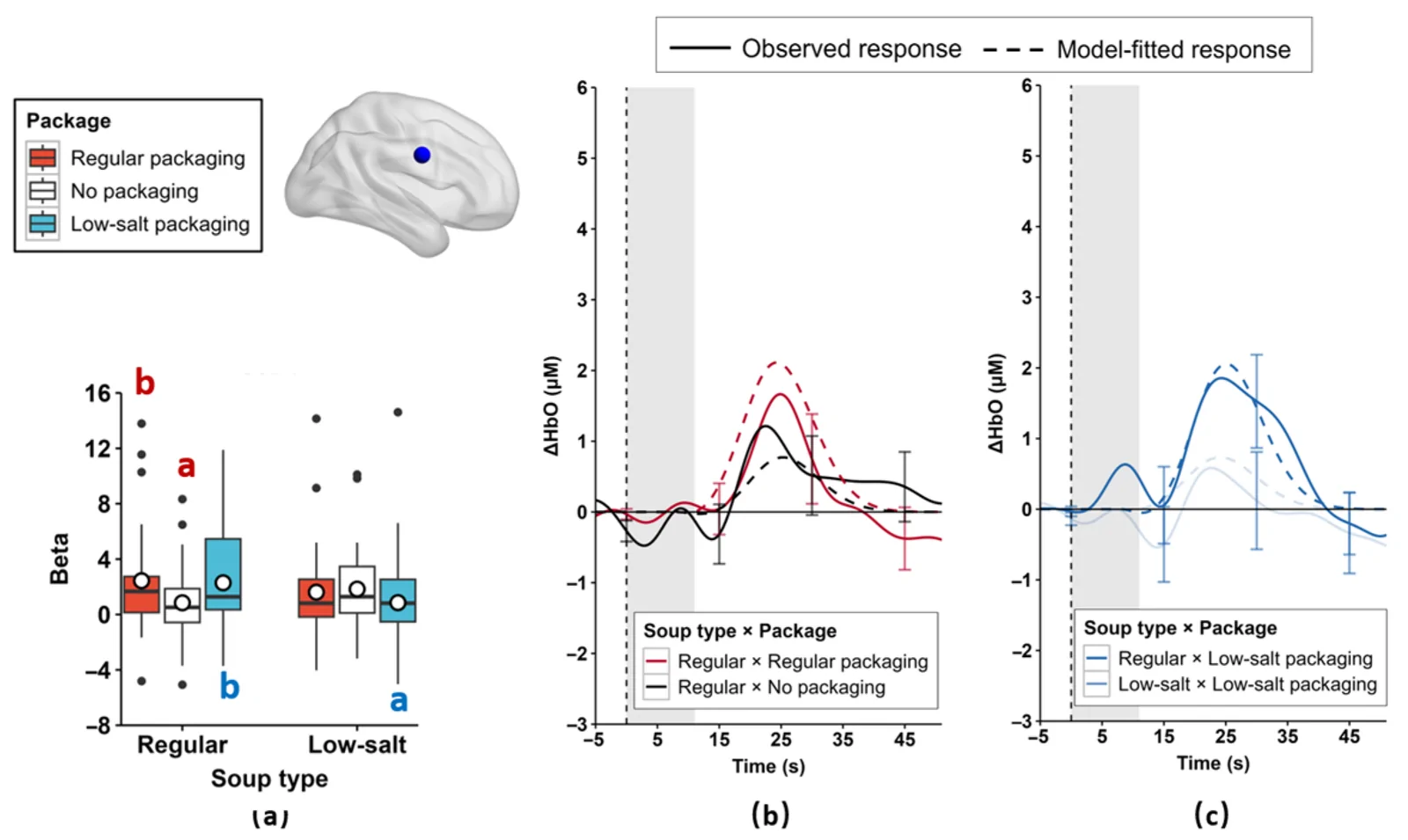
Neural response characteristics of E2-CH6 for egg soup. (**a**) Cortical location of E2-CH6 and mean beta estimates across all egg soups. The blue dot indicates the approximate cortical location of E2-CH6. Different lowercase letters of the same color above the boxes indicate statistically significant differences between conditions (adjusted *p* < 0.05). (**b**,**c**) Time-course HbO responses for pairwise comparisons: (**b**) regular packaging versus no packaging under the regular soup condition; and (**c**) regular soup versus low-salt soup under the low-salt packaging condition.

**Figure 13:**
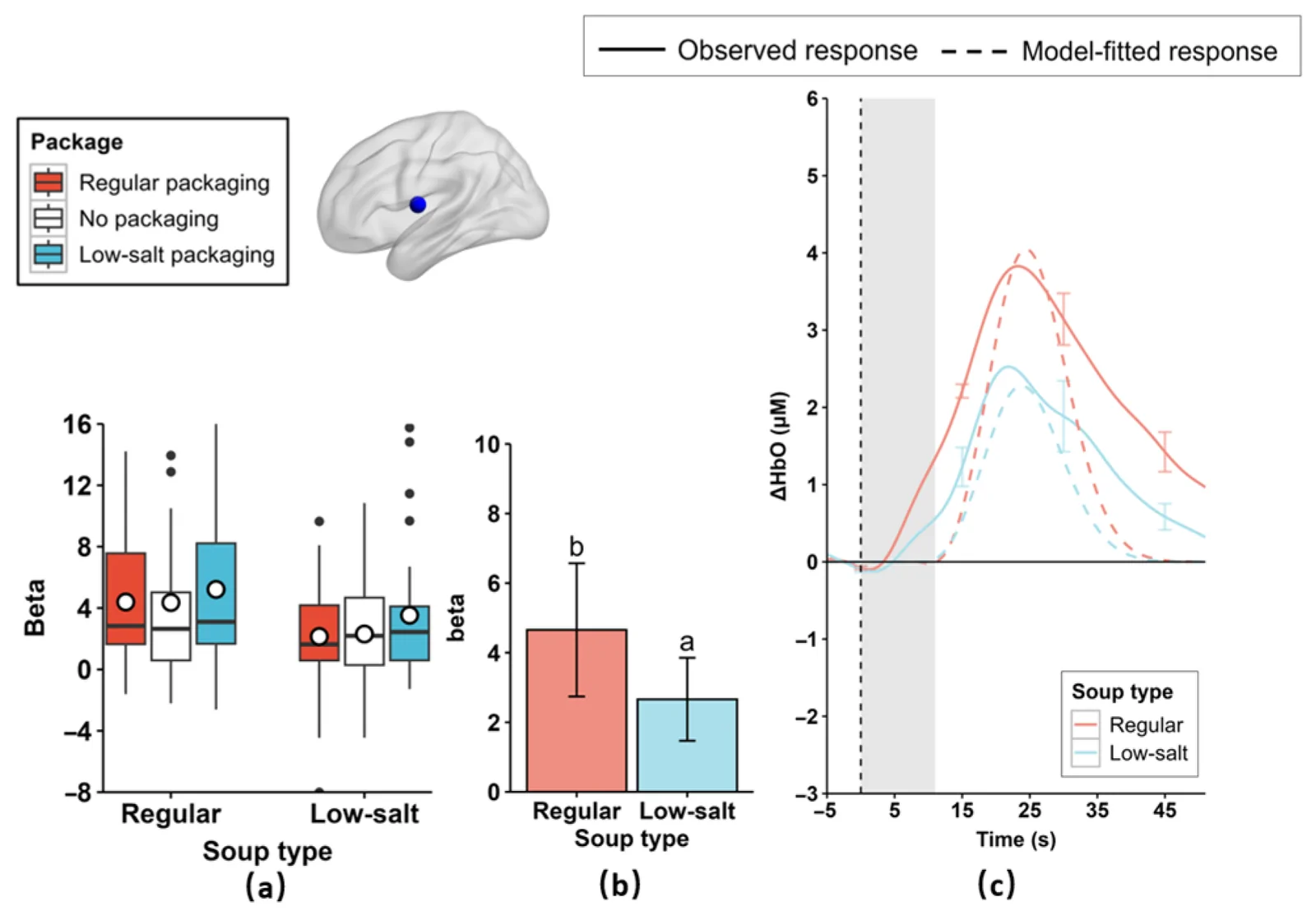
Neural response characteristics of E2-CH32 for egg soup. (**a**) Cortical location of E2-CH32 and mean beta estimates across all egg soups. The blue dot indicates the approximate cortical location of E2-CH32. (**b**) Estimated mean beta values for each soup type, collapsed across package conditions. Different lowercase letters above the bars indicate statistically significant differences between conditions (adjusted *p* < 0.05). (**c**) Time-course HbO responses for regular soup versus low-salt soup.

Exploratory post hoc comparisons at E2-CH6 (Figure 12a) showed that, under the regular soup condition, regular packaging elicited a higher response than no packaging (*t*(29) = 2.82, adjusted *p* < 0.05, 95% CI [0.200, 2.988]; Figure 12b). Under the low-salt packaging condition, regular soup elicited a higher response than low-salt soup (*t*(29) = 2.18, adjusted *p* < 0.05, 95% CI [0.089, 2.776]; Figure 12c). At E2-CH32 (Figure 13a,b), the response averaged across package conditions was higher for regular soup than for low-salt soup (*t*(29) = 3.41, adjusted *p* < 0.01, 95% CI [0.800, 3.190]; Figure 13c).

**Figure 14:**
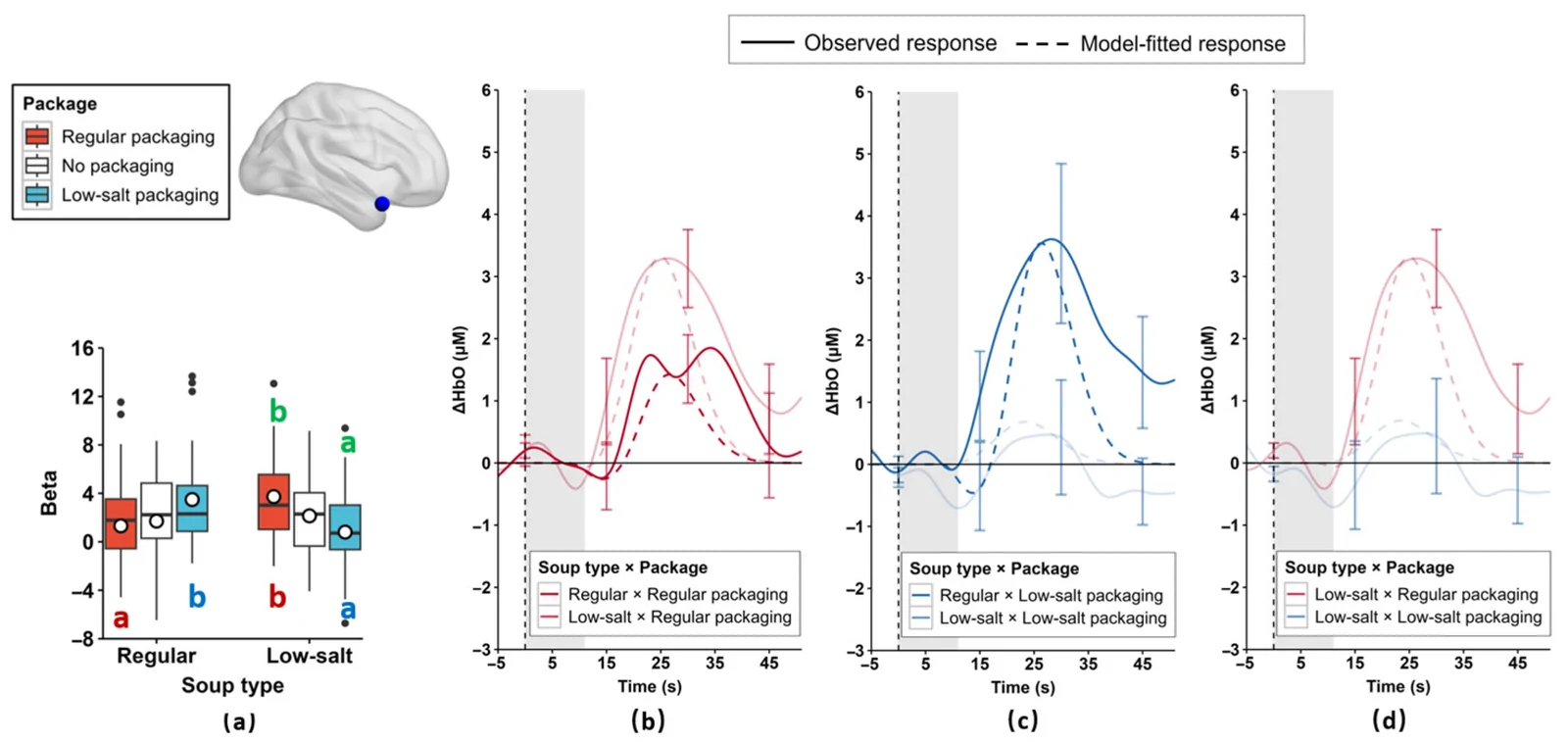
Neural response characteristics of E2-CH17 for corn soup. (**a**) Cortical location of E2-CH17 and mean beta estimates across all corn soups. The blue dot indicates the approximate cortical location of E2-CH17. Different lowercase letters of the same color above the boxes indicate statistically significant differences between conditions (adjusted *p* < 0.05). (**b**–**d**) Time-course HbO responses for pairwise comparisons: (**b**) regular soup versus low-salt soup under the regular packaging condition; (**c**) regular soup versus low-salt soup under the low-salt packaging condition; and (**d**) regular packaging versus low-salt packaging under the low-salt soup condition.

E2-CH17 (Figure 14a) showed that, with regular packaging, low-salt soup elicited a higher response than regular soup (*t*(29) = 2.07, adjusted *p* < 0.05, 95% CI [0.034, 4.792]; Figure 14b). In contrast, with low-salt packaging, regular soup elicited a higher response than low-salt soup (*t*(29) = 2.29, adjusted *p* < 0.05, 95% CI [0.288, 5.088]; Figure 14c). Additionally, under the low-salt soup condition, regular packaging elicited a higher response than low-salt packaging (*t*(29) = 2.70, adjusted *p* < 0.05, 95% CI [0.245, 5.613]; Figure 14d). Given the pattern of higher responses under mismatched packaging–soup conditions, a further post hoc contrast compared matched and mismatched conditions in E2-CH17. HbO responses were higher under mismatched conditions (*t*(29) = 3.08, *p* < 0.01, BH-adjusted *p* = 0.151, 95% CI [0.859, 4.241]), although this contrast did not survive BH correction across all 34 channels within corn soup. However, the corresponding HbR contrast showed lower responses under mismatched than matched conditions and survived the same BH correction (*t*(29) = −4.15, *p* < 0.001, BH-adjusted *p* = 0.0089, 95% CI [−2.734, −0.930]). Complete contrast results are provided in Supplementary Table S4c.

##### Channels of Interest

**Figure 15:**
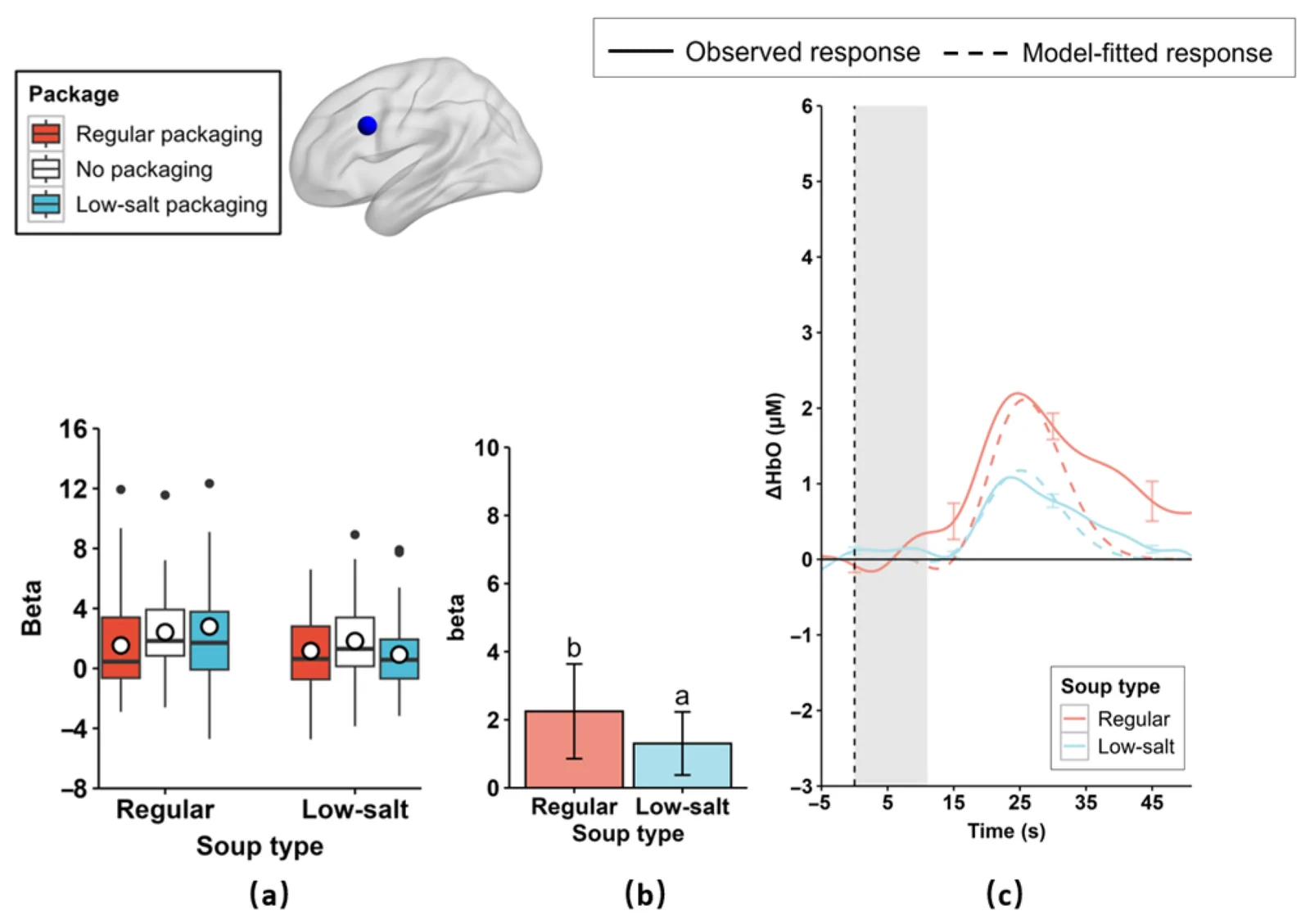
Neural response characteristics of E2-CH20 for egg soup. (**a**) Cortical location of E2-CH20 and mean beta estimates across all egg soups. The blue dot indicates the approximate cortical location of E2-CH20 (**b**) Estimated mean beta values for each soup type. Different lowercase letters above the bars indicate statistically significant differences between conditions (adjusted *p* < 0.05). (**c**) Time-course HbO responses for regular soup versus low-salt soup conditions.

For the channels of interest, E2-CH03 and E2-CH20, no significant main effects of soup type or packaging or their interaction were observed at E2-CH03 for either egg or corn soup (all *p* > 0.1; all BH-adjusted *p* ≥ 0.245). At E2-CH20 (Figure 15a), an exploratory main effect of soup type was observed for egg soup (*F*(1, 29) = 4.25, *p* = 0.048, BH-adjusted *p* = 0.163, η^2^_p_ = 0.12). Responses averaged across packaging conditions were higher for regular than low-salt soup (*t*(29) = 2.06, adjusted *p* = 0.048, 95% CI [0.008, 1.881]; Figure 15b,c). No other main effects or interactions reached significance at E2-CH20 in either soup category.

#### 3.2.3. Hemodynamic Response–Behavioral Correlation Analysis

Repeated-measures correlations were used to examine the relationships between neural responses and subjective saltiness and palatability ratings, focusing on the channels reported above. Correlations were calculated for all 34 channels that passed quality control, with BH correction applied separately for each behavioral measure within each soup category. For egg soup, the saltiness correlations at E2-CH20 (*r*_rm_ = 0.205, adjusted *p* = 0.054) and E2-CH32 (*r*_rm_ = 0.202, adjusted *p* = 0.054) did not survive correction. Among the reported channels, only E2-CH20 was significantly positively correlated with palatability (*r*_rm_ = 0.299, adjusted *p* < 0.01). No correlations survived correction for corn soup (Complete correlation results are provided in Supplementary Table S6).

## 4. Discussion

### 4.1. Experiment 1

#### 4.1.1. Behavioral Results

Experiment 1 examined whether saltiness enhancers—olfactory cues and umami substances—enhance the perceived saltiness of egg and corn soups. For egg soup, the low-salt soup with MPG + IMP had the highest mean saltiness rating among the low-salt conditions. Its mean rating fell between those of the low-salt control and regular soup, although neither comparison was significant after Tukey adjustment. The potential contribution of MPG + IMP to perceived saltiness therefore remains uncertain, and the nonsignificant difference from regular soup does not establish equivalence. For corn soup, the MPG + IMP condition and the low-salt control had nearly identical mean ratings. Reis Rocha et al. [43] reported that the saltiness-enhancing effects of umami substances varied across foods, with different umami substances showing different effects. Their findings suggest that food characteristics may be relevant when evaluating umami-based saltiness enhancement. The descriptive findings of the present study motivate further investigation of the potential contribution of MPG + IMP to saltiness perception in different food contexts. Previous studies have also suggested that congruency between umami substances and odors may influence multisensory integration. McCabe and Rolls [44] reported stronger integration in higher-order multisensory brain regions when glutamate was paired with a congruent savory odor, whereas Shepard et al. [45] reported slower detection and weaker sensory integration for an incongruent combination of MSG and odor. These findings suggest that the relationship between added umami substances and the existing flavors of a food may influence the perceptual effects of these substances. In the present study, no significant saltiness enhancement was observed, despite effects reported in previous research using NaCl solutions [13,14,15,16]. One possible explanation is that congruency between the added umami substances and the soups’ existing flavors influenced their contribution to perceived saltiness. However, congruency was not directly assessed in the present study, and this explanation therefore remains tentative.

Regarding olfactory saltiness enhancers, neither bacon nor soy sauce odor significantly increased perceived saltiness relative to the low-salt control in either egg or corn soup. Thus, the present study did not provide clear evidence of odor-induced saltiness enhancement under the tested conditions. Previous studies suggest that associations and congruency between odors and tastes may influence their combined perceptual effects [46]. Seo et al. [15] reported that, for the same NaCl solution, bacon odor enhanced perceived saltiness more strongly than strawberry odor. Dalton et al. [47] also found that congruent odor–taste combinations facilitated detection when the individual stimuli were below their respective perceptual thresholds. Together, these findings suggest that congruent odor–taste cues may be more readily integrated, potentially enhancing the perceptual salience of the overall flavor signal. In the present study, the soups’ complex flavor profiles may have influenced how the added odor cues contributed to perceived saltiness. However, this possibility was not directly assessed, and the lack of significant differences between the odor conditions and the low-salt control cannot be attributed specifically to odor–taste integration.

In terms of palatability, there were no significant differences between the low-salt and regular versions of either soup. However, the addition of soy sauce odor was associated with significantly lower palatability ratings than those for the low-salt soup condition in both soup categories. This pattern is noteworthy because, during preliminary screening, the isolated soy sauce odor was judged to have an intensity approximately comparable to that of bacon odor, and no obvious unpleasantness was noted when it was presented with the soups. The contrast between these preliminary impressions and the subsequent palatability ratings suggests that the report of no unpleasantness during preliminary screening does not necessarily imply that the added odor would leave the overall palatability of the soups unchanged. The soy sauce odor may have altered the overall flavor balance of the soups, or its intensity may have been less appropriate in the soup context. Although these possibilities remain tentative, the findings highlight the importance of considering how an added odor contributes to the overall sensory experience of the food.

The significant positive within-participant correlation between perceived saltiness and palatability for corn soup suggests that saltiness may be a relevant sensory attribute in participants’ evaluations of this food. Participants tended to find corn soup more palatable when they perceived it as saltier. For egg soup, the correlation was not significant, suggesting that higher perceived saltiness may not necessarily be accompanied by greater palatability. Other sensory attributes may also contribute to these evaluations.

Taken together, these behavioral results leave the effectiveness of MPG + IMP and the tested olfactory cues in enhancing perceived saltiness uncertain. However, the descriptive rating patterns motivate further investigation of their potential contribution to saltiness perception in foods with complex flavor profiles. Furthermore, the serial-position analysis of the three intermediate enhancer conditions for corn soup showed that saltiness ratings were higher at the fourth than at the second presentation position. This finding suggests that presentation order may have contributed to variation in saltiness ratings for corn soup.

#### 4.1.2. Hemodynamic Response Results

The results showed a condition effect on HbO responses that survived BH correction at E1-CH19 for egg soup. This channel is located approximately over the left DLPFC, with partial overlap with the premotor and supplementary motor cortices. The DLPFC has been associated with cognitive control, attentional regulation, and top-down information processing [48,49]. Recent studies have shown that this region is also involved in the cognitive aspects of taste processing. Hasegawa et al. [21] showed that activity levels in the DLPFC were significantly higher when participants chewed unfamiliar and unpleasant-tasting gum than when they chewed familiar and pleasant-tasting gum. Jiang et al. [22] reported greater left DLPFC activation during sweet solution tasting in habitual consumers of non-nutritive sweeteners. Furthermore, an fMRI study [50] showed that DLPFC activity increases when consumers process complex or unfamiliar food-label information, indicating that this region contributes to processing food-related information under conditions of increased load or uncertainty. Collectively, these studies suggest that the DLPFC is involved in cognitive aspects of food evaluation and that its responses may be influenced by unfamiliarity and prior experience. In the present study, low-salt soup elicited higher E1-CH19 responses than low-salt soup with MPG + IMP and regular soup, while regular soup elicited lower responses than low-salt soup with bacon odor. No significant correlations were detected between E1-CH19 activity and saltiness or palatability ratings. In light of previous studies [21,22,50], a tentative interpretation based on reverse inference is that the condition-related responses may involve cognitive processes similar to those discussed in these studies, although the underlying cognitive processes remain uncertain. In particular, the low-salt and regular reference soups were always presented first and fifth, respectively, so contrasts involving these conditions cannot separate condition effects from order effects. Although no significant serial-position effect was detected within positions 2–4, this supplementary analysis does not resolve the confounding involving the reference soups. Moreover, the HbR robustness analysis did not identify a corresponding significant effect. Further studies are needed to clarify whether unfamiliarity, attention, cognitive demand, or presentation order contributed to the observed responses.

E1-CH16, located approximately over the left premotor cortex, showed an exploratory condition-related difference: the MPG + IMP condition exhibited a negative HbO response that was significantly lower than that in the soy sauce odor condition. Previous fMRI research found that flavored stimuli enhanced swallowing-related responses in motor regions [51]. No significant correlations were detected between E1-CH16 responses and saltiness or palatability ratings in the present study. One possible explanation is that movements accompanying or following tasting may have differed across conditions and contributed to the measured signals. Previous studies have also suggested that negative HbO responses in motor-related regions may involve task-related movements, baseline activity, or contributions from adjacent regions [52,53]. However, the present study cannot distinguish among these potential contributions.

In Experiment 1, a main effect of soup type on HbO responses that survived BH correction was observed at E1-CH19, located approximately over the left lateral frontal cortex, with predominant overlap with BA9, a higher-order region implicated in cognitive aspects of food evaluation [21,22,50]. However, whether these responses reflected cognitive processing remains uncertain. Further studies should independently manipulate contextual cues while keeping the taste stimuli constant to examine their potential influence on food evaluation and the associated neural responses.

### 4.2. Experiment 2

#### 4.2.1. Behavioral Results

Based on the findings of Experiment 1, Experiment 2 examined whether package cues, treated as a potential cognitive factor, influenced saltiness perception and neural responses under the present experimental conditions by manipulating the match between packaging information and the actual salt content of the soups. Prefrontal regions were also selected to approximate the cortical area underlying E1-CH19 in Experiment 1, allowing further examination of responses in this area during taste evaluation.

The behavioral data showed a significant effect of soup type, but no significant effects of packaging cues on saltiness or palatability ratings. Previous studies have shown that packaging and label information can influence consumers’ taste expectations and actual taste experiences [54]. For example, Liem et al. [11] found that, in soups with different salt contents, a “low-salt” label on packaging reduced consumers’ expectations of saltiness and affected their perceived saltiness and liking. However, in another study, Liem et al. [55] found that although a “low-sodium” label significantly reduced participants’ expectations of the saltiness of chicken soup, it did not reduce their perceived saltiness. The absence of a significant labeling effect on perceived saltiness in that study is consistent with the present findings. The difference is that it remains unknown whether the package cues altered expectations in the present study. Furthermore, although the ratings varied with the actual soup type, a potential contribution of package cues to these evaluations cannot be ruled out. One possible explanation is that the regular and low-salt packages used in this study had relatively similar visual designs, which may have limited the salience of the package cues. However, because the present study did not include direct measures of expectations or a manipulation check, the contribution of cue salience to the present findings remains uncertain.

The correlation analysis showed significant positive within-participant associations between perceived saltiness and palatability for both egg and corn soups, suggesting that participants tended to rate the soups as more palatable when they perceived them as saltier in the present study.

#### 4.2.2. Hemodynamic Response Results

In Experiment 2, no main effects of soup type or package cues, or their interaction, on HbO responses survived BH correction. Effects observed at uncorrected *p* < 0.01 are therefore discussed below as exploratory findings, with interpretations treated as tentative.

For egg soup, a soup type × package interaction was observed at E2-CH6, located approximately over the Rolandic operculum. This region has been implicated in both gustatory and somatosensory processing [56] and also proposed as one of the primary gustatory regions [57]. Veldhuizen et al. [58] reported increased activity in the left dorsal mid-insula and overlying Rolandic operculum when participants attempted to detect taste in a tasteless solution compared with passively receiving the same solution. They interpreted this activation as reflecting the involvement of taste-related attentional processing, suggesting that activity in this region may be influenced not only by sensory input but also by cognitive processes such as attention to taste. In the present study, E2-CH6 showed a higher HbO response to the same regular egg soup under regular packaging than without packaging. This pattern suggests a possible association between package cues and neural responses during tasting. Under the same low-salt packaging condition, E2-CH6 showed a higher HbO response to regular soup than to low-salt soup, suggesting that its activity may also be influenced by differences in actual sensory input, including salt content. However, E2-CH6 activity was not significantly correlated with saltiness ratings. Given that the Rolandic operculum has also been implicated in both gustatory and lingual somatosensory processing [56,57], its response may reflect multiple aspects of the tasting experience rather than perceived saltiness alone. Overall, these exploratory findings suggest a possible association of neural responses at E2-CH6 with both packaging conditions and the sensory properties of the soups.

For egg soup, an exploratory main effect of soup type was observed at CH32, with a higher HbO response to regular soup than to low-salt soup. This channel is located approximately over the superior temporal gyrus (STG), which is widely involved in auditory and language processing [59]. However, previous studies also reported its involvement in taste-related processing. Kinomura et al. [60] observed STG activation in a task requiring participants to distinguish between saltwater and pure water and suggested that this region may form part of a neural network involved in taste discrimination and monitoring. Using EEG, Tzieropoulos et al. [61] found that higher salt concentrations elicited early taste-related responses with greater amplitude and shorter latency, with source localization implicating the STG among the contributing regions. In this study, E2-CH32 activity showed only a weak correlation with saltiness ratings. The higher response to regular soup may involve sensory or task-related processes accompanying its evaluation.

For corn soup, E2-CH17, located approximately over the temporal pole, showed an exploratory soup type × package interaction in HbO responses, while the corresponding HbR interaction survived BH correction. A direct post hoc contrast between matched and mismatched soup type–packaging conditions was also conducted, with BH correction across all channels. This contrast showed higher HbO and lower HbR responses under mismatched conditions, but only the HbR difference survived correction, suggesting a possible sensitivity to soup type–packaging mismatch. As part of the higher-order associative cortex, the temporal pole is a critical region for transforming external perceptual inputs into higher-level semantic representations [62,63]. Mesulam [64] further argued that the temporal pole contributes to the integration of past experiences, contextual information, and expectations, thereby triggering corresponding emotional, interoceptive, and evaluative responses. Wilton et al. [65] observed an N400 event-related potential associated with expectation violation or semantic incongruence when the perceived sweetness intensity did not match the expected intensity indicated by the label. In the present study, CH17 activity was not significantly correlated with either saltiness or palatability ratings, suggesting that its response may not be closely related to these subjective evaluations. Based on the studies discussed above, a possible interpretation is that the response pattern under mismatched conditions may involve processing the discrepancy between packaging information and actual taste input.

Regarding the channels of interest, E2-CH3 and E2-CH20, only E2-CH20 showed a main effect of soup type for egg soup at uncorrected *p* < 0.05. This effect did not survive BH correction and is therefore discussed as exploratory. McClure et al. [31] showed that brand information altered neural responses to the same cola, including activity in the hippocampus and DLPFC. The authors suggested that taste evaluation involves not only sensory information but also higher-order processes such as memory and cognitive control, which may contribute to preference judgments. E2-CH20 activity showed a modest positive correlation with palatability ratings that survived BH correction, indicating that higher activity tended to accompany higher palatability ratings. Considered alongside this association, the stronger response to regular soup may be partly related to hedonic evaluation during tasting.

Overall, in Experiment 2, no significant effects of package cues on saltiness or palatability ratings were observed. Although no main effects or interactions in HbO responses survived BH correction, exploratory findings provided preliminary evidence of neural response differences associated with soup type and package cues. In particular, E2-CH17 showed a possible sensitivity to soup type–packaging mismatch, with additional support from the BH-corrected HbR interaction and matched–mismatched contrast. However, given the limited penetration depth of fNIRS, measurements are primarily restricted to superficial portions of some regions, limiting coverage of regions such as the temporal pole and Rolandic operculum discussed here. Signals recorded over temporal regions, particularly at E2-CH17 near the temporalis muscle, may also be affected by jaw movement, swallowing, temporalis muscle activity [66], and extracerebral hemodynamics. These findings provide a basis for further investigation of packaging-related processing during tasting through improved control of potential signal confounds and the integration of complementary measurement methods.

### 4.3. General Discussion

The behavioral results of Experiment 1 did not provide clear support for the saltiness enhancement proposed in H1. Under the present experimental conditions, saltiness enhancers previously shown to be effective in NaCl solutions [13,14,15,16] did not significantly enhance perceived saltiness in soups with more complex flavor profiles. Although the MPG + IMP condition had the highest mean saltiness rating among the low-salt egg soup conditions, its difference from the low-salt soup condition was not statistically significant. The differing patterns in saltiness ratings between egg and corn soups motivate further investigation of the role of food flavor context proposed in H2, but do not establish food-dependent enhancement. For the neural results, the main effect of soup type on HbO responses at E1-CH19 survived BH correction for egg soup. In light of previous studies, a tentative interpretation based on reverse inference is that the higher response to low-salt soup may involve cognitive aspects of taste evaluation, possibly related to unfamiliarity or attentional demands. However, the fixed presentation positions of the low-salt and regular soups confound condition effects with presentation-order effects. Furthermore, cognitive factors such as familiarity and congruence were not directly assessed. The present findings therefore cannot establish whether the observed differences reflect specific cognitive mechanisms.

Experiment 2 yielded findings relevant to H3, but did not establish the hypothesized effects. For the behavioral data, no significant effects of package cues on saltiness or palatability ratings were detected. Regarding the neural data, although no HbO effects survived BH correction, exploratory analyses revealed response differences across conditions. In particular, the response pattern at E2-CH17 suggested possible sensitivity to package–soup type mismatch. These findings provide a basis for further investigation of packaging-related processing during food evaluation, although the underlying cognitive processes remain to be clarified.

Prefrontal responses were observed in both Experiments 1 and 2, although their statistical support differed, with the E2-CH20 finding remaining exploratory. E1-CH19 and E2-CH20 were both located approximately over the DLPFC but did not necessarily sample identical cortical areas. Responses were higher to low-salt than regular egg soup in Experiment 1, whereas the opposite pattern was observed in Experiment 2. No significant correlation with palatability was detected at E1-CH19, while E2-CH20 showed a modest positive correlation. These observations motivate further investigation of how contextual information and hedonic evaluation relate to prefrontal responses during tasting.

In addition, despite the use of the same soup stimuli, no significant difference in palatability between low-salt and regular soups was detected in Experiment 1, whereas a significant difference was observed for both soup categories in Experiment 2. One possible interpretation is that the different evaluation contexts and presentation procedures influenced participants’ judgements. The range of sensory conditions in Experiment 1 and the packaging conditions in Experiment 2 may have provided different frames of reference for evaluating the soups. Presentation order may also have influenced this pattern. These possibilities warrant further investigation.

### 4.4. Limitations

In Experiment 1, the congruency between the added sensory cues and the food flavor profile was not directly assessed. Previous studies have suggested that congruency can influence olfactory–gustatory integration and related perceptual effects [15,26,30,45]. However, explicitly asking participants to evaluate congruency before or during tasting could have directed their attention toward the relationship between the sensory components and potentially influenced subsequent sensory judgments. Therefore, congruency was not directly assessed in the present study, and its contribution to the observed effects that depend on the flavor profile remains unclear. Future studies specifically designed to examine olfactory–gustatory congruency in foods with complex flavor profiles are required to clarify its role. Another limitation in Experiment 1 is that the low-salt soup was presented first to provide a common initial reference for all participants. However, because the presentation positions of the low-salt and regular soups were fixed, the condition effects could not be fully separated from possible effects of order of presentation, which may have biased the evaluations. As the present study focused mainly on participants’ initial responses to taste conditions, future taste studies using fewer sample conditions and repeated stimulus presentations may further examine and control for order-related effects. Lastly, to minimize the influence of additional sodium ions on saltiness perception, this study used MPG to examine the saltiness-enhancing effect of umami in soups. However, this also introduced additional potassium ions, which may have affected the taste of the soups. Among the soups used in this study, only the low-salt egg soup provided a labeled potassium content of 143 mg per serving. Based on this value, the estimated potassium content per tasting sample increased from approximately 19 mg to 36 mg after the addition of MPG, nearly doubling the original amount. In concentration terms, this corresponded to an increase from approximately 0.095% to 0.18% (w/v), with the final concentration remaining relatively low. The addition of IMP also introduced a small amount of sodium; however, this amounted to only approximately 2.4–6.1% of the sodium originally present in the soups. Nevertheless, the potential effects of these added ions on the overall taste of the soups cannot be ruled out. Further experiments are needed to clarify whether and to what extent these ions affected taste.

In Experiment 2, because package cues were presented before the soup stimuli, they may have induced salt-related taste expectations to some extent and influenced subsequent responses. Future research could measure participants’ expectations of saltiness before tasting to clarify the relationship between expectations elicited by packaging information, taste perception, and neural responses.

From a methodological perspective, short-separation channels and additional physiological monitoring were not available in this study. Although the timing of participants’ actions was controlled across experimental conditions and the fNIRS data were preprocessed using filtering and artifact-correction procedures, potential contributions from hand and mouth movements, respiration, and other systemic physiological fluctuations could not be fully excluded. These factors may therefore have partially contributed to the observed fNIRS signals. In addition, although the sample size was determined based on an a priori power analysis for the behavioral repeated-measures design, the power analysis did not specifically account for the fNIRS channel-wise analyses and multiple comparisons. Therefore, the statistical power for detecting neural effects may have been limited.

Further research should examine how experimental paradigms and food-related characteristics shape the cognitive demands of participants and sensory evaluations. Because taste and smell signals are often ambiguous, participants may rely on task instructions, evaluation criteria, external information, and intrinsic food characteristics when interpreting sensory inputs. Factors such as flavor profiles, familiarity, and baseline saltiness may therefore need to be considered more systematically. Clarifying how these factors interact with experimental design to guide participants’ attention and evaluation bias will be important for interpreting neural responses related to sensory processing.

## 5. Conclusions

Taken together, neither the tested saltiness enhancers nor package labels significantly increased the perceived saltiness of the soups under the present experimental conditions. The descriptive findings from Experiment 1 motivate further investigation of the potential role of food flavor context, while the neural findings from Experiment 2 suggest possible condition-related responses, including potential sensitivity to package–soup type mismatch. These findings encourage further investigation of sensory and cognitive factors across different food and presentation contexts to inform the development of low-salt foods.

## Figures and Tables

**Table 1:** Experimental conditions and composition of soup stimuli.

Soup Category	Salt Content	Soup Powder Content	Water	Saltiness Enhancers	Amounts	Soup Type
Egg soup	1.2 g (Regular)	6.5 g	160 mL	-	-	Regular
	0.7 g (Low-salt)	6.5 g	160 mL	-	-	Low-salt
				MPG + IMP	0.66 g + 0.19 g	Low-salt + MPG + IMP
				Bacon odor	20 μL	Low-salt + Bacon
				Soy sauce odor	5 μL	Low-salt + Soy
Corn soup	1.0 g Regular	18.6 g	150 mL	-	-	Regular
	0.48 g (Low-salt)	18.6 g	150 mL	-	-	Low-salt
				MPG + IMP	0.62 g + 0.18 g	Low-salt + MPG + IMP
				Bacon odor	20 μL	Low-salt + Bacon
				Soy sauce odor	5 μL	Low-salt + Soy

## Data Availability

The complete dataset, including the raw and processed data supporting the findings of this study, is publicly available from the Open Science Framework (OSF) repository at https://doi.org/10.17605/OSF.IO/USY8X.

## Supplementary Materials

The following supporting information can be downloaded at: https://www.mdpi.com/article/10.3390/foods15213916/s1. Figure S1: Presentation-order allocation in Experiment 1; Figure S2: Channel-wise HbO beta estimates for egg soup in Experiment 1; Figure S3: Channel-wise HbO beta estimates for corn soup in Experiment 1; Figure S4: Channel-wise HbO response time courses for egg soup in Experiment 1; Figure S5: Channel-wise HbO response time courses for corn soup in Experiment 1; Figure S6: Channel-wise HbO beta values for egg soup in Experiment 2; Figure S7: Channel-wise HbO beta values for corn soup in Experiment 2; Figure S8: Channel-wise HbO response time courses for egg soup in Experiment 2; Figure S9: Channel-wise HbO response time courses for corn soup in Experiment 2. Table S1: Channel localization in Experiment 1; Table S2: Channel localization in Experiment 2; Table S3a,b: Complete HbO and HbR statistical results for Experiment 1; Table S4a–c: Complete HbO and HbR statistical results for Experiment 2, including matched–mismatched contrasts; Table S5: Complete neural–behavioral correlation results for Experiment 1; Table S6: Complete neural–behavioral correlation results for Experiment 2.

## Abbreviations

The following abbreviations are used in this manuscript:

AL	Left preauricular point
ANOVA	Analysis of variance
AR	Right preauricular point
BA6	Brodmann area 6; premotor cortex and supplementary motor area
BA9	Brodmann area 9; dorsolateral prefrontal cortex
BA44	Brodmann area 44; pars opercularis
BA45	Brodmann area 45; pars triangularis
Cz	Vertex
DLPFC	Dorsolateral prefrontal cortex
EEG	Electroencephalography
fMRI	Functional magnetic resonance imaging
fNIRS	Functional near-infrared spectroscopy
GLM	General linear model
IMP	Disodium inosinate
Iz	Inion
MPG	Monopotassium glutamate
MSG	Monosodium glutamate
NaCl	Sodium chloride
Nz	Nasion
SD	Standard deviation
STG	Superior temporal gyrus
TI	Time-intensity
