# Supplementary material for: Sensory and Cognitive Modulation of Saltiness Processing in Everyday Food: Evidence from Two fNIRS Experiments
Source: Foods. 2026 Nov 1;15(21):3916. doi: 10.3390/foods15213916 (PMC13762865; doi:10.3390/foods15213916)
Supplement: Supplementary file 1 [file foods-15-03916-s001.zip › Supplementary_Data_Figures.pdf]

A. Block order of Soup

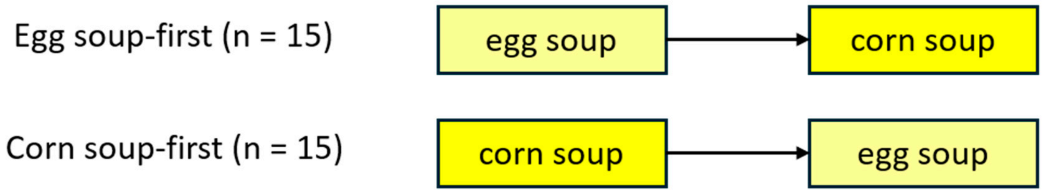

B. Stimulus presentation pattern

Pattern	Position 1	Position 2	Position 3	Position 4	Position 5	Participants IDs (Egg first)	Participants IDs (Corn first)
1	Low-salt	U	S	B	Regular	1, 2, 25	13, 14, 28
2		U	B	S		3, 4, 26	15, 16, 29
3		S	U	B		5, 6, 27	17, 18, 30
4		S	B	U		7, 8	19, 20
5		B	S	U		9, 10	21, 22
6		B	U	S		11, 12	23, 24

U: MPG + IMP; S: Soy sauce odor; B: Bacon odor

C. Condition frequencies at positions 2-4

	Position 2	Position 3	Position 4
**U: MPG + IMP**	12	10	8
**S: Soy sauce odor**	10	10	10
**B: Bacon odor**	8	10	12

The six intermediate patterns were not equally frequent

Figure S1. Stimulus presentation patterns in Experiment 1. (A) Fifteen participants completed the egg-soup block first and 15 completed the corn-soup block first. (B) Low-salt and regular soups occupied positions 1 and 5, respectively. The same intermediate pattern was used in both blocks for each participant. (C) Condition frequencies at positions 2–4 were identical for egg and corn soups.

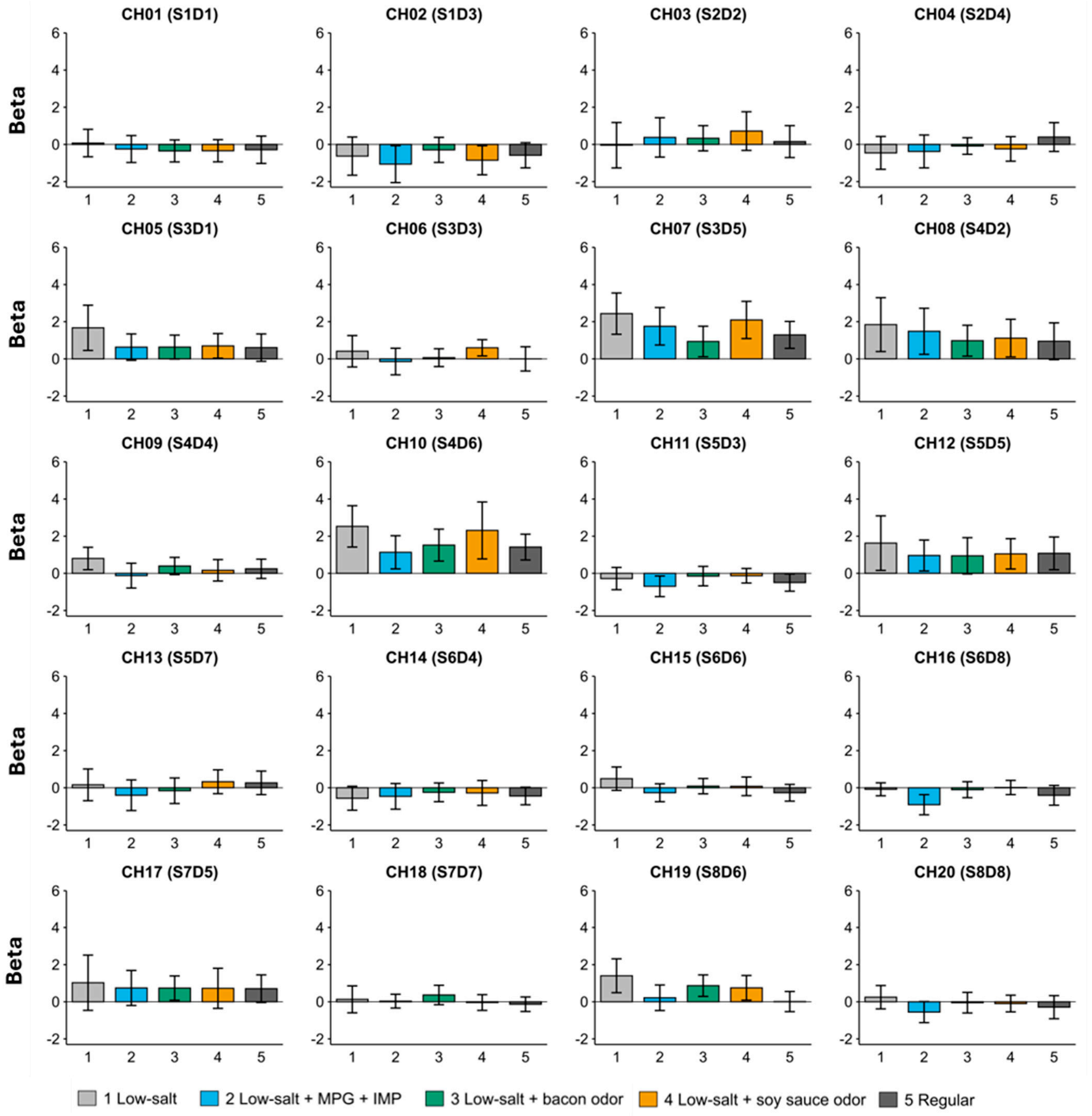

Figure S2. Channel-wise HbO beta estimates for egg soup in Experiment 1. Bars show estimated marginal means; error bars show pointwise 95% confidence intervals.

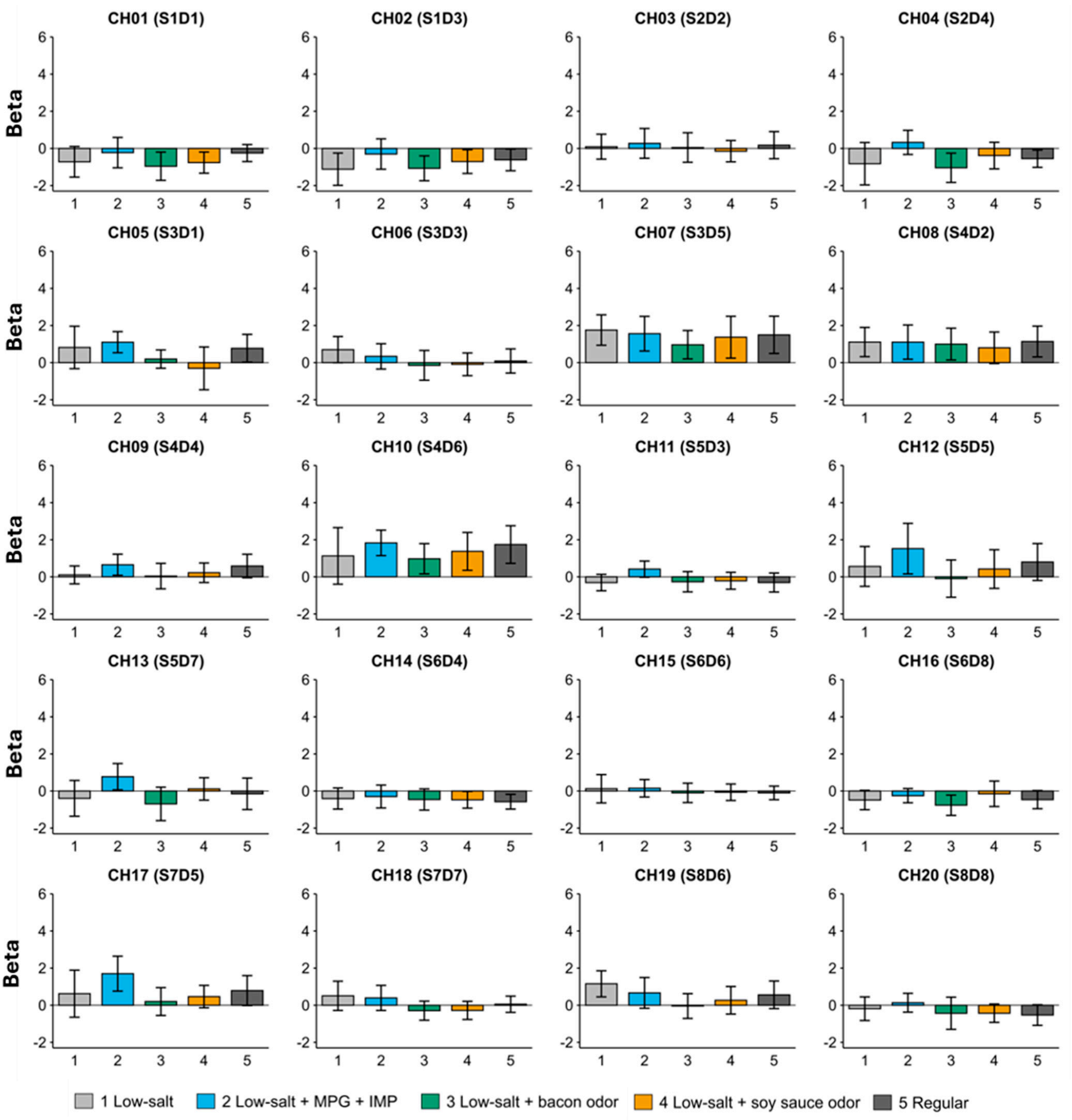

Figure S3. Channel-wise HbO beta estimates for corn soup in Experiment 1. Bars show estimated marginal means; error bars show pointwise 95% confidence intervals.

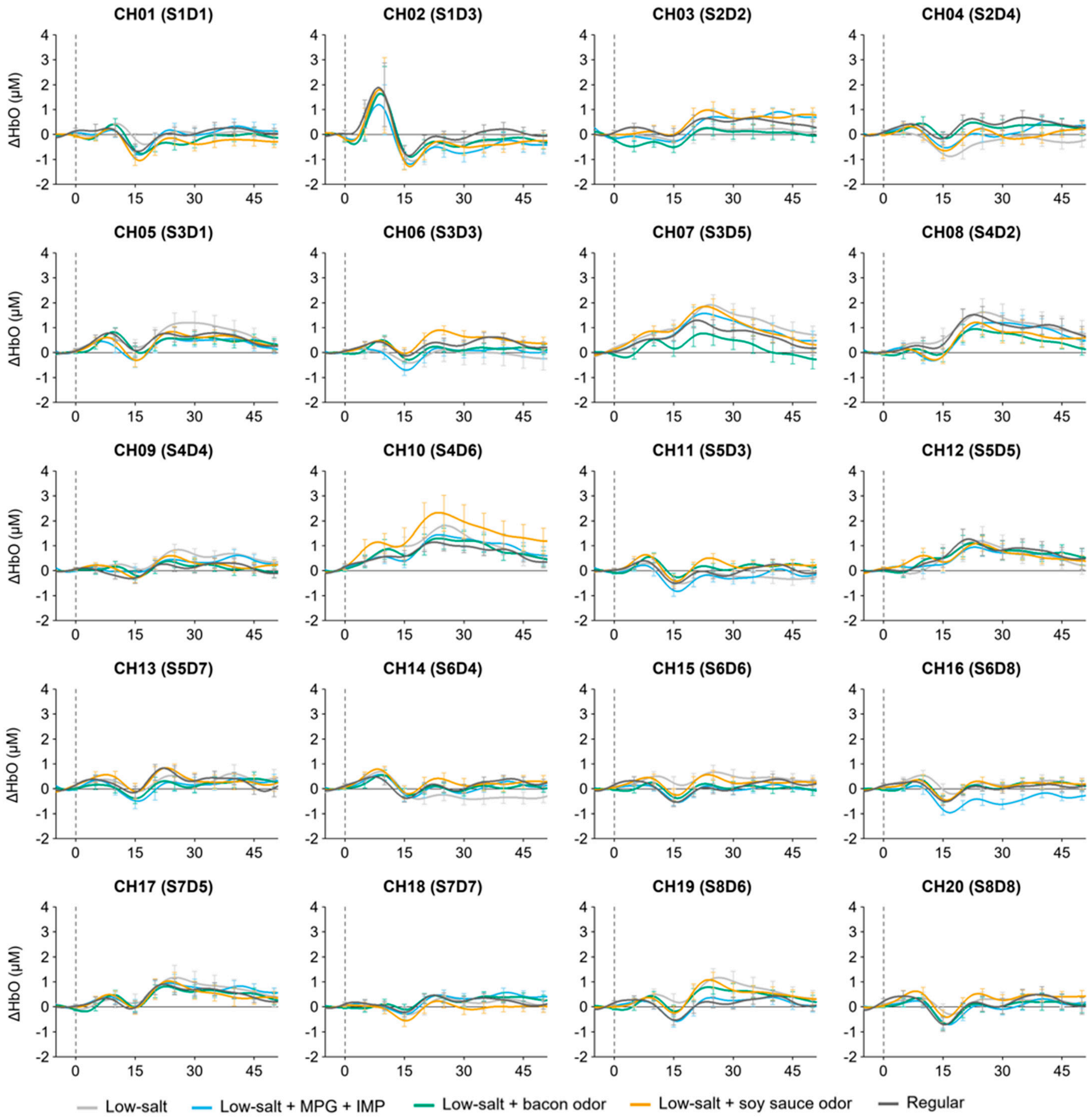

Figure S4. Channel-wise HbO response time courses for egg soup in Experiment 1. Lines show mean responses; error bars indicate standard errors.

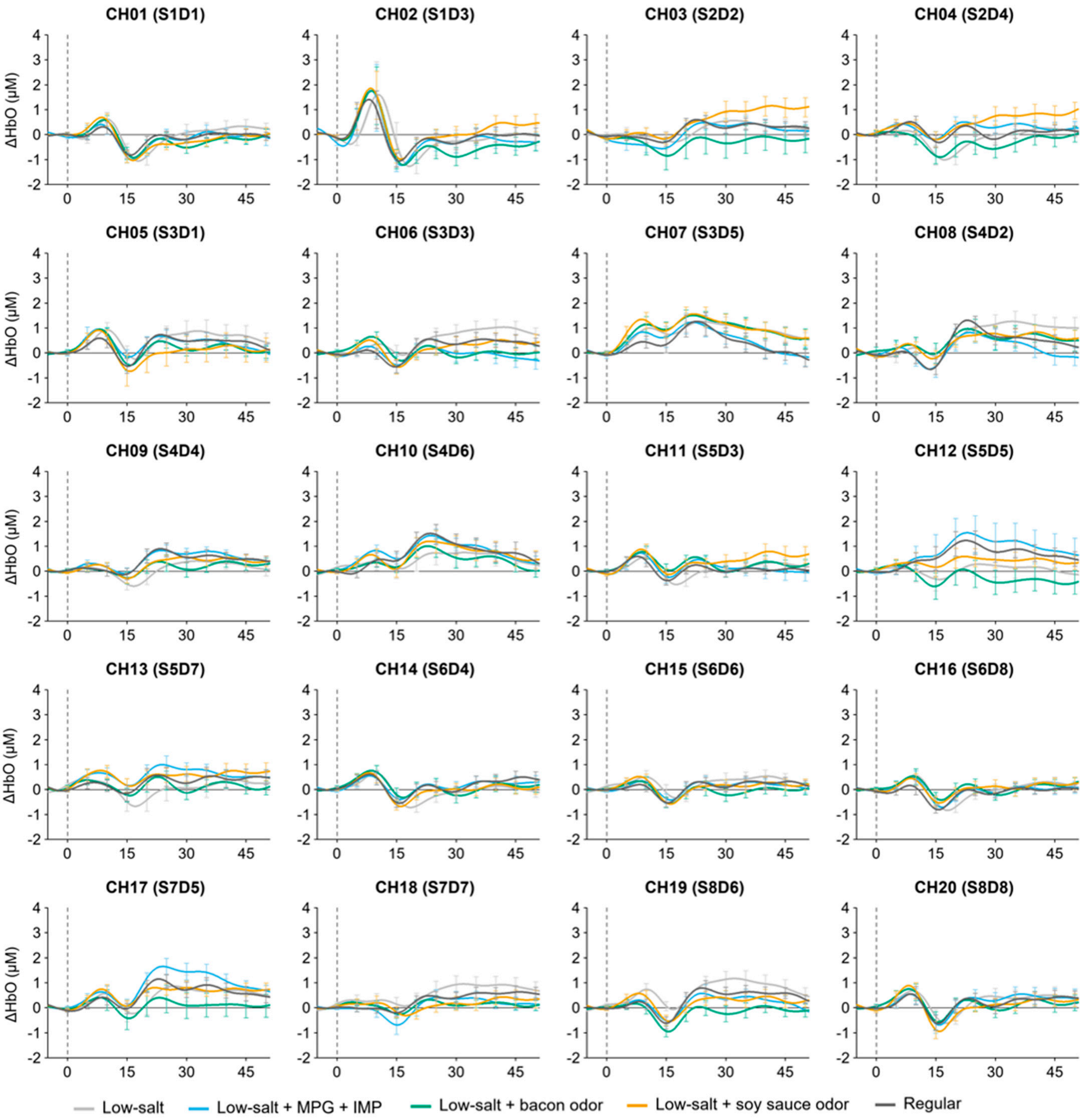

Figure S5. Channel-wise HbO response time courses for corn soup in Experiment 1. Lines show mean responses; error bars indicate standard errors.

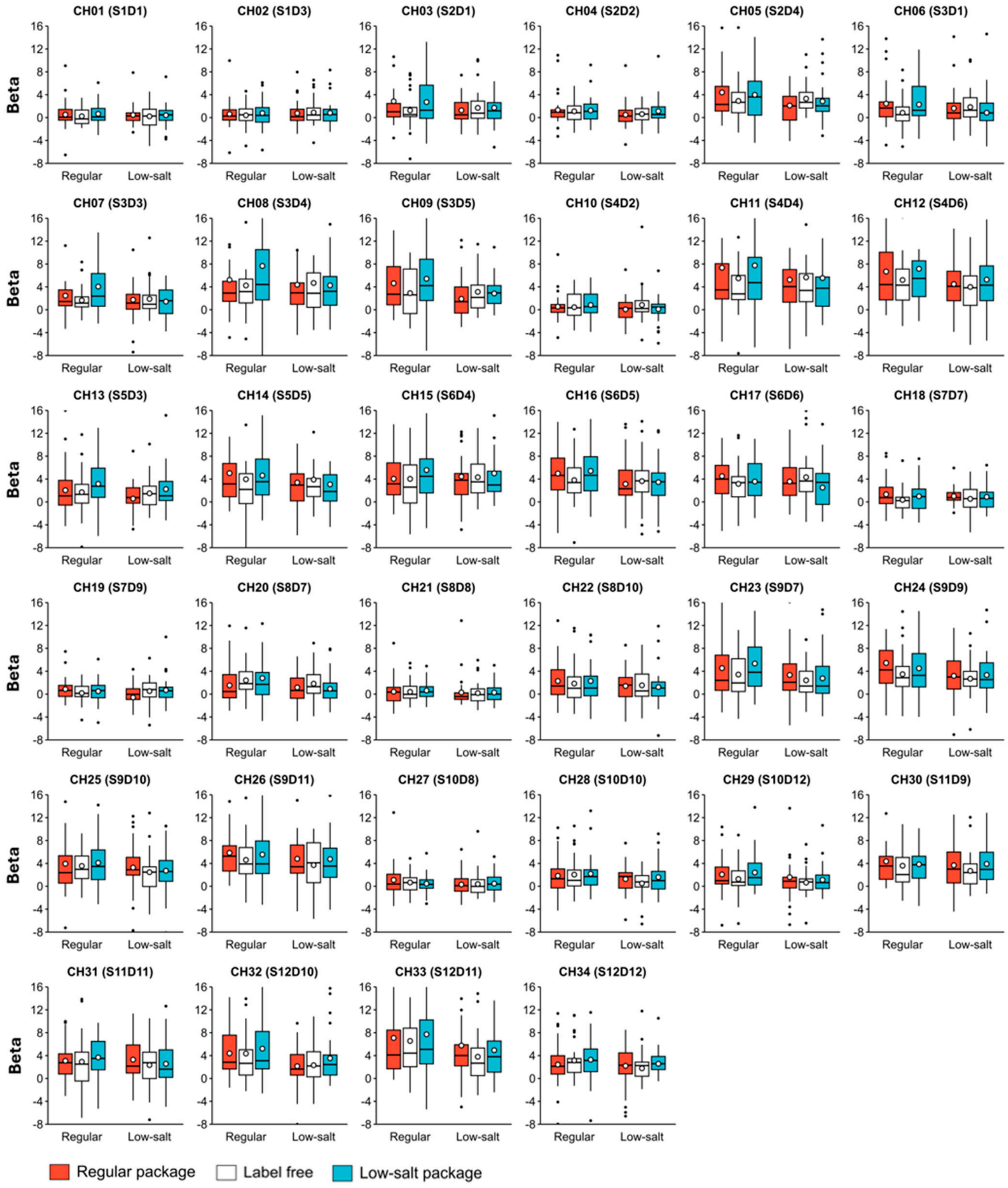

Figure S6. Channel-wise HbO beta values for egg soup in Experiment 2. Boxes show medians and interquartile ranges. Black dots indicate outliers, and white circles indicate means.

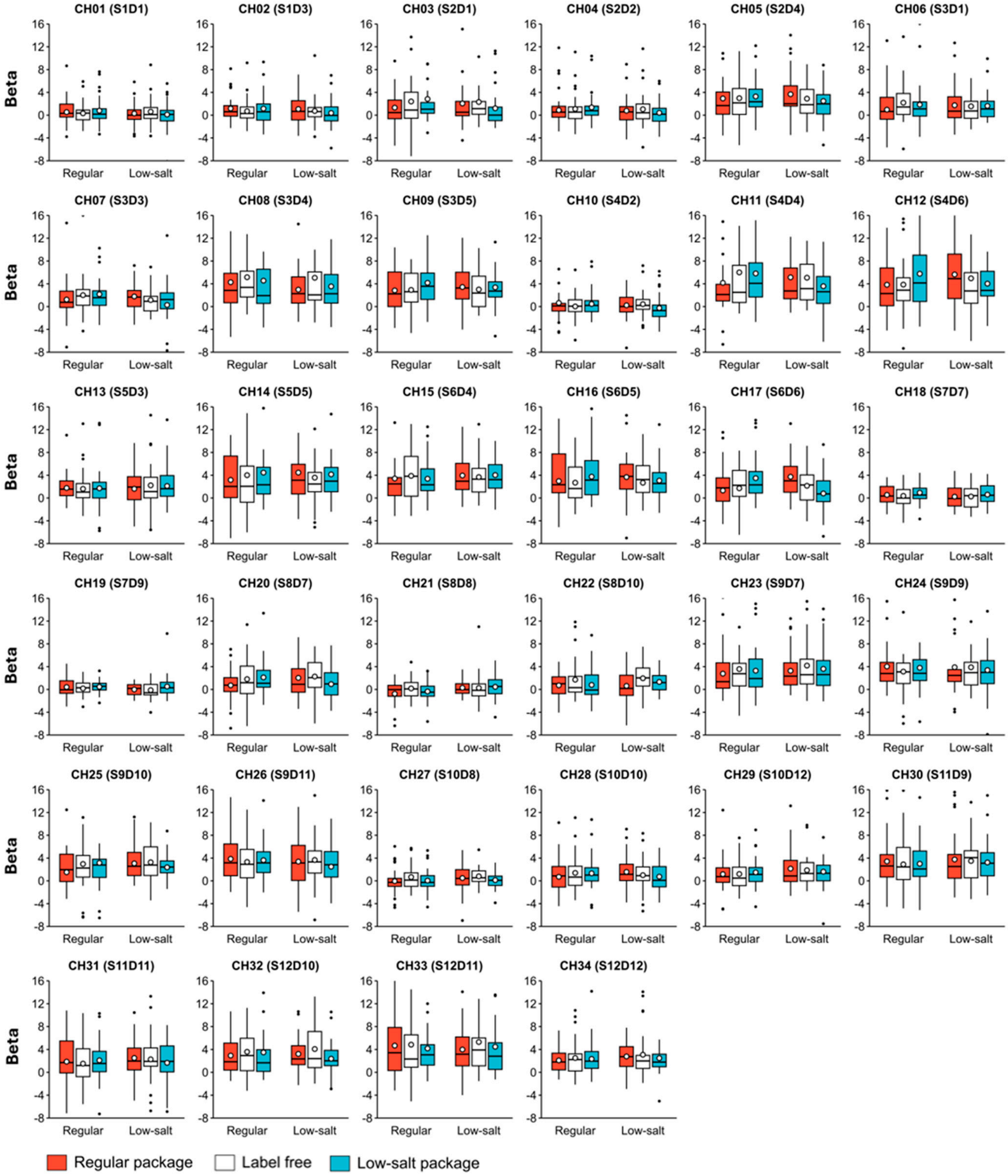

Figure S7. Channel-wise HbO beta values for corn soup in Experiment 2. Boxes show medians and interquartile ranges. Black dots indicate outliers, and white circles indicate means.

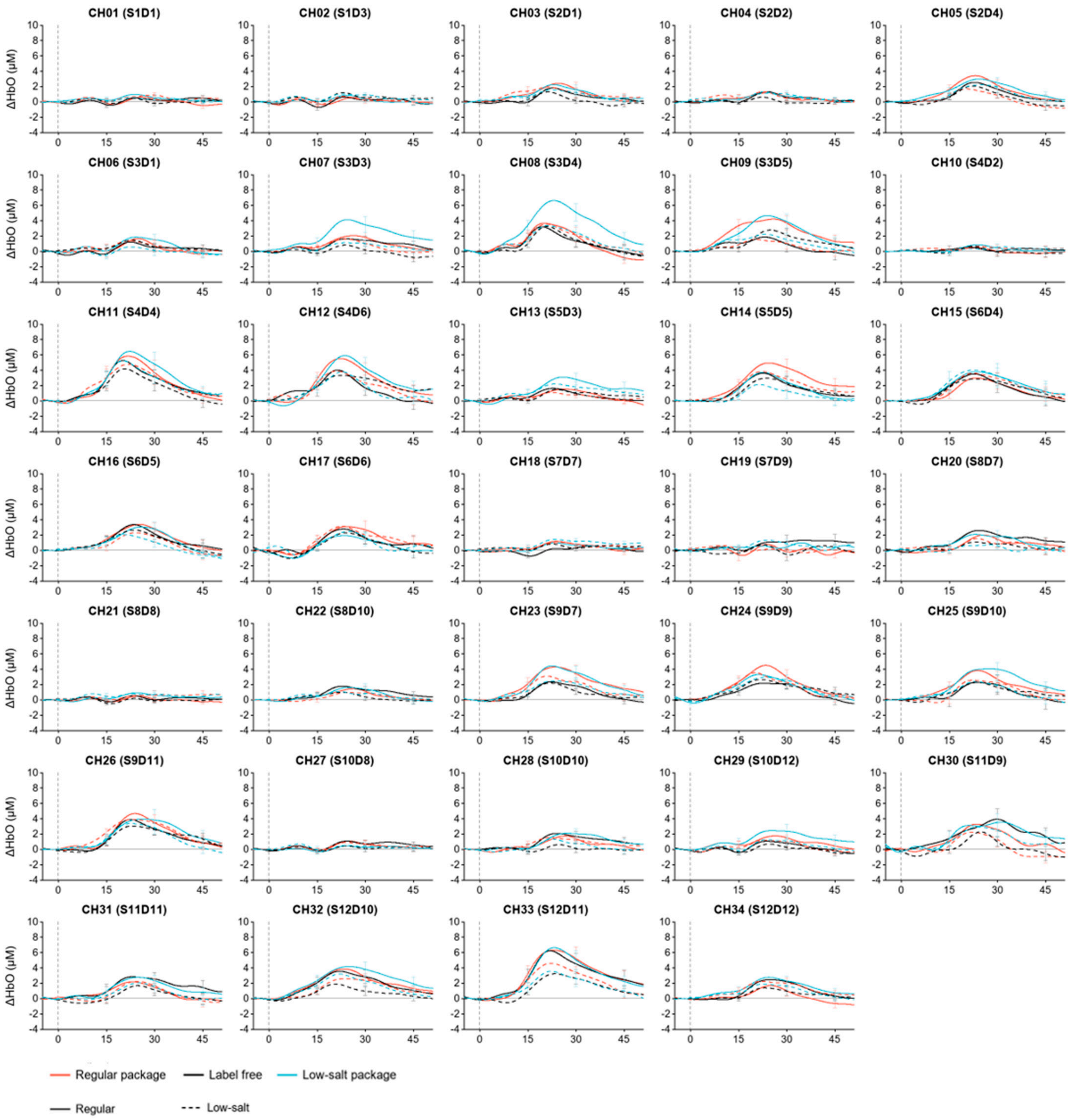

Figure S8. Channel-wise HbO response time courses for egg soup in Experiment 2. Lines show mean responses; error bars indicate standard errors.

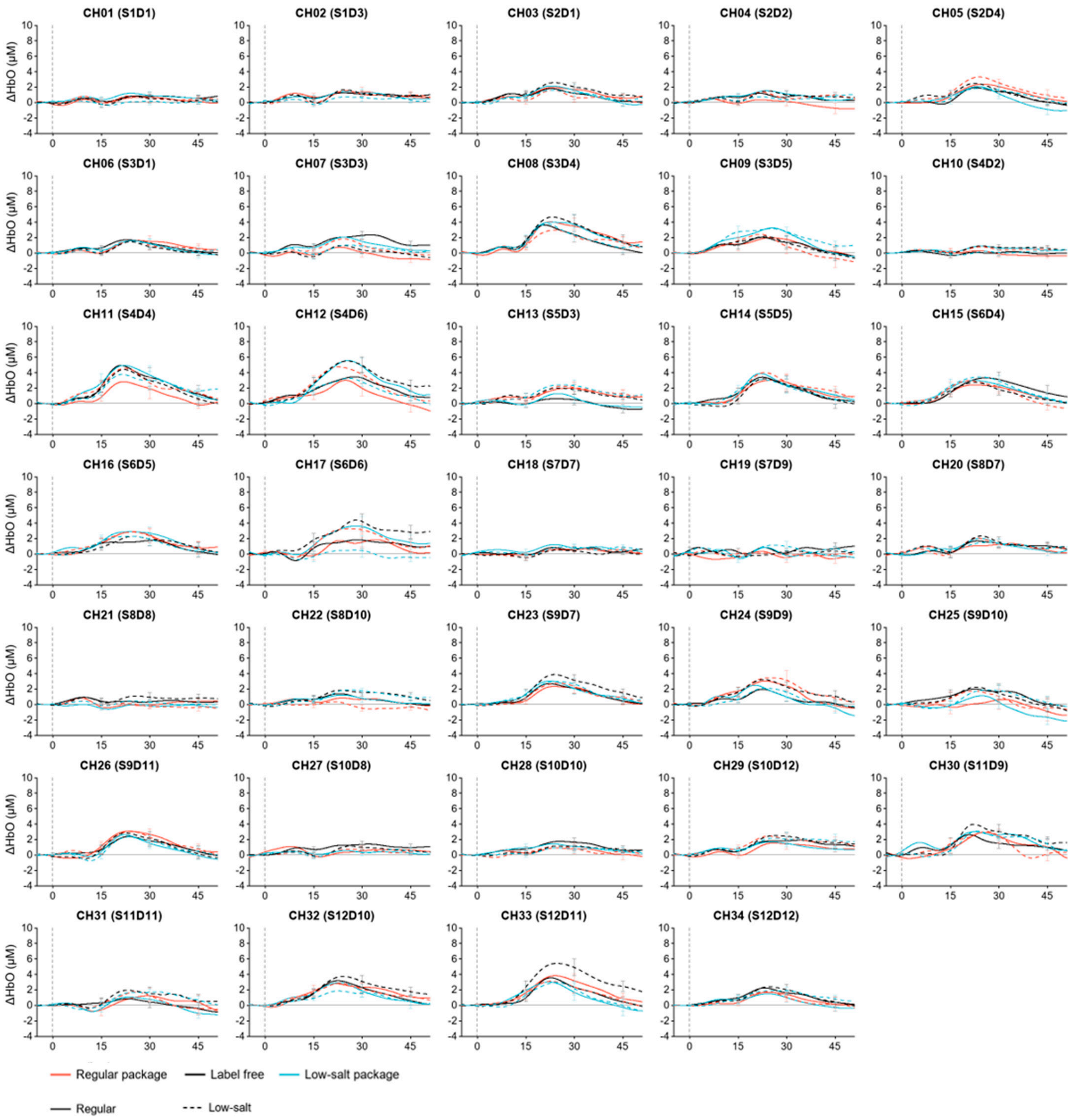

Figure S9. Channel-wise HbO response time courses for corn soup in Experiment 2. Lines show mean responses; error bars indicate standard errors.
